# Limited ATF4 Expression in Degenerating Retinas with Ongoing ER Stress Promotes Photoreceptor Survival in a Mouse Model of Autosomal Dominant Retinitis Pigmentosa

**DOI:** 10.1371/journal.pone.0154779

**Published:** 2016-05-04

**Authors:** Yogesh Bhootada, Pravallika Kotla, Sergei Zolotukhin, Oleg Gorbatyuk, Zsuzsanna Bebok, Mohammad Athar, Marina Gorbatyuk

**Affiliations:** 1 Department of Optometry and Vision Science, University of Alabama at Birmingham, Birmingham, Alabama, United States of America; 2 Vision Science Research Center, University of Alabama at Birmingham, Birmingham, Alabama, United States of America; 3 Department of Pediatrics, University of Florida, Gainesville, Florida, United States of America; 4 Center for Neurodegeneration and Experimental Therapy, University of Alabama at Birmingham, Birmingham, Alabama, United States of America; 5 Department of Cell, Developmental and Integrative Biology, University of Alabama at Birmingham, Birmingham, Alabama, United States of America; 6 Department of Dermatology, University of Alabama at Birmingham, Birmingham, Alabama, United States of America; Indiana University College of Medicine, UNITED STATES

## Abstract

T17M rhodopsin expression in rod photoreceptors leads to severe retinal degeneration and is associated with the activation of ER stress related Unfolded Protein Response (UPR) signaling. Here, we show a novel role of a UPR transcription factor, ATF4, in photoreceptor cellular pathology. We demonstrated a pro-death role for ATF4 overexpression during autosomal dominant retinitis pigmentosa (ADRP). Based on our results in ATF4 knockout mice and adeno-associated viral (AAV) delivery of ATF4 to the retina, we validated a novel therapeutic approach targeting ATF4 over the course of retinal degeneration. In T17M rhodopsin retinas, we observed ATF4 overexpression concomitantly with reduction of p62 and elevation of p53 levels. These molecular alterations, together with increased CHOP and caspase-3/7 activity, possibly contributed to the mechanism of photoreceptor cell loss. Conversely, ATF4 knockdown retarded retinal degeneration in 1-month-old T17M Rhodopsin mice and promoted photoreceptor survival, as measured by scotopic and photopic ERGs and photoreceptor nuclei row counts. Similarly, ATF4 knockdown also markedly delayed retinal degeneration in 3-month-old ADRP animals. This delay was accompanied by a dramatic decrease in UPR signaling, the launching of anti-oxidant defense, initiation of autophagy, and improvement of rhodopsin biosynthesis which together perhaps combat the cellular stress associated with T17M rhodopsin. Our data indicate that augmented ATF4 signals during retinal degeneration plays a cytotoxic role by triggering photoreceptor cell death. Future ADRP therapy regulating ATF4 expression can be developed to treat retinal degenerative disorders associated with activated UPR.

## Introduction

T17M rhodopsin mice, carrying a mutant human rhodopsin (*RHO*) gene, have become a valuable murine model for studying autosomal dominant retinitis pigmentosa (ADRP). Severe retinal degeneration is detected by one month in these animals, resulting in a significant loss of scotopic a- and b-wave ERG amplitudes and in compromised retinal integrity [1–3]. This is primarily due to apoptotic photoreceptor cell death. The mechanism of retinal degeneration in the ADRP retina includes the impaired assembly of the opsin protein with its chromophore, 11-cis-retinal, and is due to rhodopsin modified stability, folding, and trafficking [4]. The accumulation of mutant, misfolded rhodopsin in the endoplasmic reticulum (ER) triggers a signal transduction cascade known as the Unfolded Protein Response (UPR), which we have described earlier in T17M *RHO* photoreceptors [3]. In these mice we observed that PERK (RNA-activated protein kinase-like ER kinase) signaling, is upregulated leading to an increase in phosphorylated (p) translational eukaryotic initiation factor 2α (eIF2α) and ATF4 (Activating transcription factor 4 or cAMP responsive element binding protein, CREB) mRNA, starting at postnatal day (P) 18. The accumulation of peIF2α has also been found in the retina of another ADRP rat model carrying the P23H *RHO* gene [5]. This mutant, like the T17M RHO mutant, represents a Class II mutation [6] and leads to dramatic retinal degeneration [5]. These findings suggest that activated PERK signaling could be an underlying hallmark of progressive ADRP.

One of the major mediators of PERK signaling is the transcription factor ATF4. ATF4 plays a pivotal role both in adaptation to cellular stress and apoptosis. Activation of the PERK pathway induces phosphorylation of eIF2α, an upstream activator of ATF4. ATF4 in turn regulates the expression of the apoptosis-related CHOP transcription factor [7]. In addition, an extracellular stress signal is known to be promoted by ATF4-controlled expression of growth factors, cytokines, chemoattractants and adhesion molecules [8].

ATF4 is also known to be crucial to many other physiological activities including hematopoiesis, lens and skeletal development, learning and memory formation, hypoxia resistance, tumor growth, autophagy, and amino acid deprivation [9]. The role of ATF4 in promoting neurodegenerative disorders, however, is ambiguous and therefore still under investigation. It is known that ATF4 may exert either protective or deleterious effects on cell survival, depending on the paradigm [10, 11]. The benefits from ATF4 overexpression or deficit are a source of continuous debate in the literature. ATF4 is expressed constitutively at low levels but becomes rapidly induced under particular cell-stress conditions. Elevated expression of ATF4 results in its binding to the promoter regions of target genes that are involved in amino acid metabolism, redox control, and apoptosis [12, 13].

In ocular degenerative diseases, the elevation of ATF4 as well as the activation of the UPR have been described in different retinal cell types, reviewed in [14], however, the involvement of ATF4 in the pathology of diabetes [15], oxygen-induced retinopathy [16, 17] and retinitis pigmentosa [3, 5, 18] remains unclear.

Here, we have unraveled the role of ATF4 in the progression of autosomal dominant inherited retinopathy. We have demonstrated for the first time that ATF4 overexpression accelerates retinal degeneration whereas genetic ablation of ATF4 mitigates retinal degeneration and significantly protects ADRP photoreceptors from rapid deterioration in ATF4^+/-^ mice. The results of this study together with our earlier work [3, 7, 18] highlight PERK signaling as a cellular network underlying retinal degeneration. Our data also validate ATF4 as a potential therapeutic target for retarding retinal degeneration.

## Materials and Methods

### Animals

All experiments with mice followed the animal protocol approved by the Animal Care and Use Committee of the University of Alabama at Birmingham, conforming to the Association for Research in Vision and Ophthalmology guidelines. All experimental procedures were carried out in accordance with the Institutional Animal Care and Use Committee (IACUC) protocol (Approval Number # 121109792). Transgenic mice expressing human T17M rhodopsin, C57BL6/J, RHO^-/-^ and ATF4^+/-^ knockout mice were used in this study to generate T17M RHO^+/-^ (T17M) and T17M RHO^+/-^ ATF4^+/-^ (T17M ATF4^+/-^) carrying mutant and endogenous mouse rhodopsins. These mice were compared with control groups of C57BL6/J and ATF4^+/-^ mice, expressing two copies of endogenous mouse rhodopsin. All the mice had the same genetic background as C57BL/6J mice. ATF4^+/-^ mice were purchased from Jackson (Bar Harbor, ME; http://www.jax.org) and have a normal appearance, organ morphology and eye development.T17M RHO mice were created as described [19]. All mice were raised under a 12-h dim light/12-h dark cycle with access to a standard diet and tap water ad libitum. The mice were housed five per cage, and their physiological condition was monitored daily by veterinary and care staff. An euthanasia protocol based on intraperitoneal injections of ketamine and xylazine was used in the study for mice to minimize pain and distress. At the end of the study, mice were sacrificed using overdose of ketamine and xylazine followed by cervical dislocation.

The genotyping of ATF4^+/-^ mice was performed using forward primers: ATATTGCTGAAGAGCTTGGCGGC for Neo allele and AGCAAAACAAGACAGCAGCCACTA for wild type allele and a common reverse primer GTTTCTACAGCTTCCTCCACTCTT for both alleles. The T17M genotyping was performed as previously described [1]. To visualize UPR activation and splicing of the Xbp1 transcriptional factor (IRE1 pathway) in the ADRP retina, ERAI (**ER** stress **A**ctivating **I**nducer) mice were used as previously described [3].

Sub-retinal injections were performed in pups at postnatal day 15 with 1 μl of either AA2/5 virus (serotype 5) expressing the mouse ATF4 cDNA or GFP (10^13^ genome particle per ml for both viruses) driven by the CMV enhancer-chicken β-actin (CBA) promoter in their right eye and GFP in their left eye, as previously described [3, 20]. Animals were monitored for 2 weeks. ERG and protein analysis of injected mice were performed to evaluate the results of ATF4 overexpression.

### Electroretinography

Mice were dark-adapted overnight, then anesthetized with ketamine (100 mg/kg) and xylazine (10 mg/kg), and their pupils were dilated in dim red light with 2.5% phenylephrine hydrochloride ophthalmic solution (Akorn, Inc.). Scotopic ERGs were recorded using a wire contacting the corneal surface with 2.5% hypromellose ophthalmic demulcent solution (Akorn.Inc). ERG was performed at different light intensities (0 db (2.5 cd*s/m2), 5 db (7.91 cd*s/m2), 10 db (25 cd*s/m2), and 15 db (79.1 cd*s/ m2). Five scans were performed and averaged at different light intensities. Photopic, cone-mediated responses were performed following 10 min light adaptation on the background light intensity of 30 cd*s/m2. Recordings were obtained at the light intensity of 25 cd*s/m2. Fifteen waveforms from each animal were recorded and the values were averaged. The a-wave amplitudes were measured from the baseline to the peak in the cornea-negative direction, and the b-wave amplitudes were determined from the cornea-negative peak to the major cornea-positive peak. The signal was amplified, digitized, and stored using the LKC UTAS-3000 Diagnostic System (Gaithersburg, MD).

### Spectra Domain-Optical Coherent Tomography (SD-OCT)

SD-OCT was performed in P30, P60 and P90 animals using the Spectral Domain Ophthalmic Imaging System (SDOIS) (Bioptigen). The mice were anesthetized. Horizontal volume scans through the area dorso-temporal from the optic nerve (superior retina) and the area ventro-temporal from the optic nerve (inferior retina) were used to evaluate the thickness of the ONL. For measuring the thickness of the ONL, six calibrated calipers were placed in each region of the superior and inferior hemispheres of retinas within 100, 200, 300 and 400 μm of the optic nerve head. The thickness of the ONL was determined by averaging six measurements.

### Retinal Imaging

Mice were anesthetized with 100 mg/kg ketamine and 10 mg/kg xylazine intraperitoneally, and the pupils were dilated using 2.5% phenylephrine hydrochloride ophthalmic solution (Akorn, Inc.). The fundus was examined using the Micron IV camera (Phoenix Research Laboratories, Pleasanton, CA) with StreamPix 5 software in fluorescent modes. GFP fluorescence was detectable using green fluorescent barrier filters.

### Histology and Immunohistochemistry

Mouse eyes were enucleated at 1 and 3 months of age and were fixed overnight in 4% paraformaldehyde freshly made in phosphate-buffered saline (PBS) as previously described [1]. Hematoxylin and eosin staining of 12-micron retinal cryosections as well as counting of retinal nuclei were performed as previously described [2]. Digital images of right and left retinas of individual mice were analyzed in the central superior and inferior equally located from the optic nerve head. A masked investigator analyzed the images.

For immunohistochemistry retinal cryosections were rinsed in PBS and blocked in 2% normal goat serum, 0.3% Triton X-100 in 0.01% BSA in PBS for 1 hour at room temperature. The sections were then stained with primary anti-rhodopsin antibodies (1D4, University of British Columbia, Vancouver, Canada) which were diluted in PBS with 0.1% Triton X-100 and 1% BSA and incubated overnight at 4°C. The Cy2-labeled anti-IgG secondary antibody (Jackson ImmunoResearch Laboratories, West Grove, PA) diluted 1:500 in PBS was applied at room temperature for 1 hour. Sections were mounted with Vectashield Mounting Medium (Vector Lab) and cover slipped. Images were taken using a confocal microscope (Leica SP1 UV Confocal Laser Scanning Microscope).

For staining retinas for reactive oxygen species, 12-micron sections were incubated with a general oxidative stress indicator, CM-H2DCFDA, Invitrogen (10 μM/L in PBS) in the dark for 1 hour. Sections were washed three times and were mounted and coverslip with Vectashield Mounting Medium. The 485nM fluorescence filter was used to take images. Images were converted to black and white to improve contrast.

### Quantitative real-time RT-PCR

For RNA extraction, whole retinas were isolated from 1- month-old mice (C57BL6/J, ATF4^+/-^, T17M and T17M ATF4^+/-^) by surgical excision. Total RNA was extracted using the QIAGEN RNeasy Mini Kit. One μg of purified RNA was reverse transcribed into cDNA using iScriptTM Reverse Transcription Supermix (BioRad). Integrity of the RNA samples as well as efficiency of cDNA reaction was verified prior to the qRT-PCR. TaqMan Gene Expression Assay kits (Applied Biosystems) were used to measure gene expression (mRho: Mm00520345m1; HRho: Hs00892431_m1; Gapdh: Mm99999915g1; Bip: Mm00517690_g1; XBP1: Mm03464496_m1; GADD34: Mm00435119_m1; CHOP: Mm01135937_g1). Quantitative real-time PCR was performed with the Step One PlusTM Real-Time PCR System (Applied Biosystems) based on the relative standard curve method. Reactions were performed at 50°C for 2 minutes and 95°C for 10 minutes, followed by 40 cycles at 95°C for 15 seconds and 60°C for 1 minute. Results were expressed as cycle threshold time (Ct) and were normalized to Ct times for the housekeeping gene GAPDH. The replicated RQ (Relative Quantity) values for each biological sample were averaged. Biological samples from each strain were used for qRT-PCR analyses.

### Western Blot

For protein extraction, whole retinas were isolated from 1- month-old mice (C57BL6/J, ATF4^+/-^, T17M and T17M ATF4^+/-^) by surgical excision. Total protein was extracted via sonication in a protein extraction buffer containing 25 mM sucrose, 100 mM Tris-HCl, pH = 7.8, and a mixture of protease inhibitors (PMSF, TLCK, aprotinin, leupeptin, and pepstatin). Protein concentrations were determined using BioRad Protein Assays based on the Bradford method for protein quantitation. Proteins (30–40 μg) were separated in 4–20% Criterion Precast gels and 5% polyacrylamide gels (BioRad), transferred to a polyvinylidene difluoride (PVDF) membrane using the Trans-Blot Turbo Transfer System (BioRad) and incubated with primary antibodies overnight at 4°C under agitation. After washing, the membranes were incubated for 1.5 h with respective (anti-mouse, anti-rabbit or anti-goat immunoglobulin) Fluorescence or HRP conjugated secondary antibody (LI-COR Odyssey). For HRP tagged antibody blots were developed with enhanced chemiluminescence (ECL) according to manufacturer’s instructions (Amersham Bioscience). β-actin was used as a gel loading control and was detected using an anti-β-actin antibody (1:5000, Sigma-Aldrich, #A1978). The developed membrane was imaged using the LI-COR Odyssey Quantitative Fluorescence Imaging System. For list of primary antibodies and dilutions see S1 Table.

### Caspase 3/7 Activity Assay

The detection of caspase 3/7 activity was performed using the Caspase-Glo 3/7 Luciferase assay (#G8091; Promega, Madison, WI) kit in accordance with the manufacturer's recommendations. Luciferase signal was read by a luminescent plate reader (Infinite m200, Tecan Group Ltd., Meannedorf, Switzerland) and used to compare the activation of caspase 3/7 in C57BL6/J, ATF4^+/-^, T17M and T17M ATF4^+/-^retinal tissues.

### Statistical analysis

Two-way ANOVA comparisons were used to calculate differences in the a- and b-wave ERG amplitudes and in the average ONL thickness of inferior and superior retinas in 1-, 2- and 3-month-old mice. A one-way ANOVA was used to calculate a fold change of mRNA expressions and a level of normalized proteins in P30 retinas. For all experiments, *P*-value lower than 0.05 was considered significant (*P<0.05, **P<0.01, ***P<0.001, and ****P<0.0001). Data are represented using mean ± SEM.

## Results

### Overexpression of ATF4 in the T17M retina accelerates retinal degeneration

**Fig 1:**
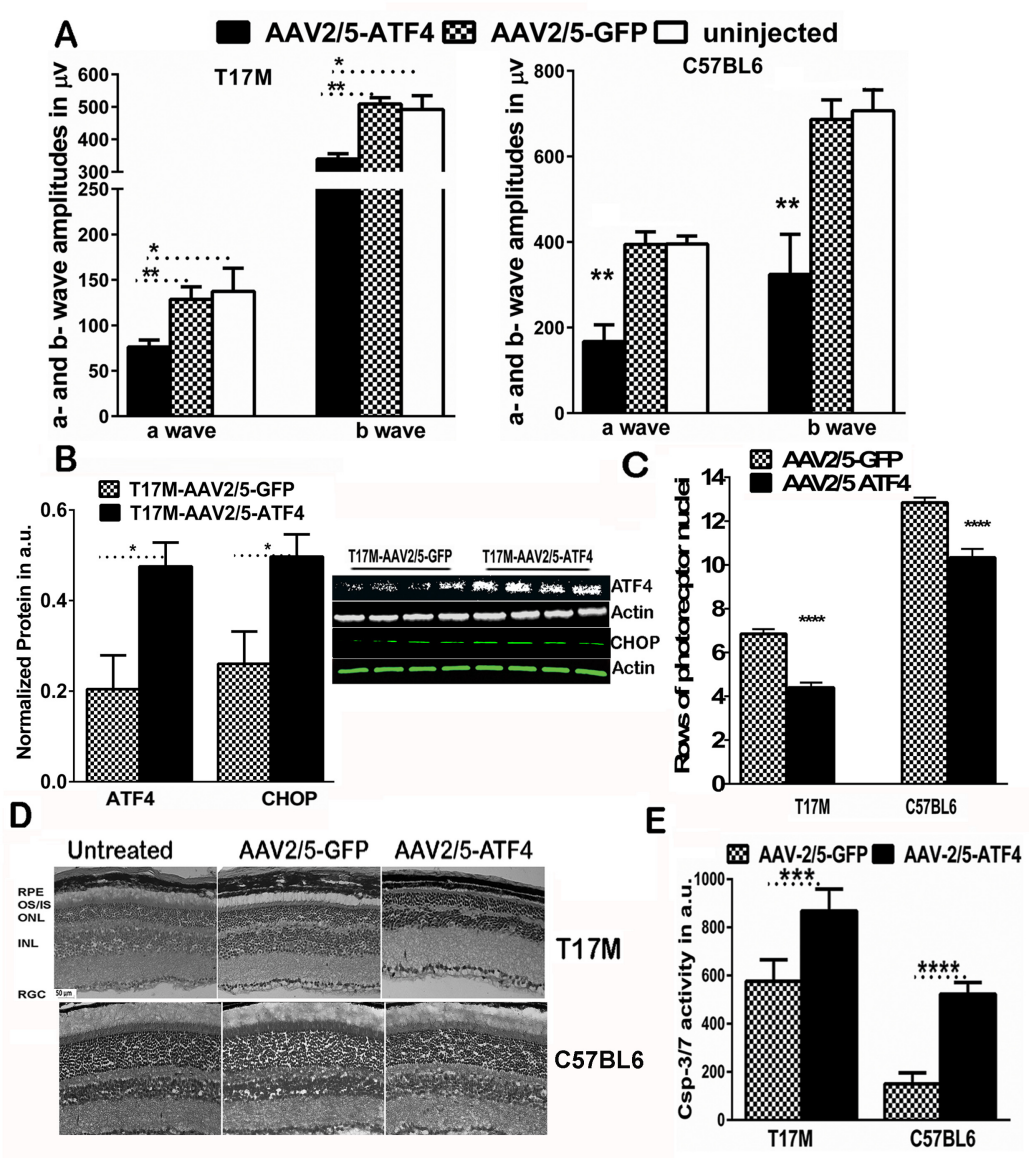
Overexpression of ATF4 accelerates and induces retinal degeneration in T17M mice and C57BL6 mice, respectively. **(A**) ERG amplitudes were registered as described in the methods and were analyzed by one-way ANOVA. Data analysis demonstrated that subretinal injections of AAV2/5- ATF4 led to a loss of photoreceptor function in the right ATF4-injected T17M retina compared to the left AAV2/5- GFP-injected eye (N = 6, *P*<0.05 for both a and b-wave amplitudes). The C57BL6 retina overexpressing ATF4 also experienced a decline in a- and b- wave ERG amplitudes (N = 4, for both *P*<0.01) as compared to GFP-injected controls or uninjected animals (N = 4). Results of the scotopic ERG amplitudes registered at 25 cd*s/m2 are shown. **(B)** Overexpression of ATF4 in the injected (right) retinas was measured by western blotting. A 2.3-fold (N = 6, *t*-test p = 0.017) increase in ATF4 was found in the right eye when compared to the left eye. This resulted in upregulation of CHOP protein (N = 6, *t*-test p = 0.046) in ATF4 overexpressing eyes. Representative images of western blots probed with antibody against ATF4, CHOP, and β-actin are shown on the side. **(C)** Overexpression of ATF4 and CHOP proteins in T17M and C57BL6 retinas was associated with a loss of photoreceptor cells measured by counting the nuclei rows in H&E stained retinal sections (N = 4, *t*-test *P*<0.001 and N = 3, *t*-test *P*<0.001). **(D)** Representative images of the H&E stained T17M and C57BL6 retinas injected with AAV2/5-GFP and AAV2/5-ATF4. **(E)** Photoreceptor cell death in T17M retinas overexpressing ATF4 was associated with highly activated caspase-3/7. The activation of apoptotic cell death markers was also detected in the wild type retinas over-expressing ATF4 (N = 4, *t-*test *P*<0.001 and *P*<0.0001, respectively). The data are presented as mean ± SEM. See also S1 Table.

To demonstrate the effect of ATF4 overexpression in ADRP retinas, we injected P15 T17M and C57BL6 pups with AAV2/5- ATF4 in the right and AAV2/5-GFP in the left eyes. This serotype has been shown to preferentially transduce photoreceptors in the retina [21, 22] and the viral-mediated GFP expression in the eye was confirmed by Micron IV funduscopy (S1 Fig). The physiological response of photoreceptors to ATF4 overexpression was measured by scotopic ERG at 2-weeks post-injection. This time point was chosen since only a short window of opportunity is available for functional and structural rescue in T17M mice, which experience photoreceptor cell death by P30 (S1 Fig). Our analysis of ERG recordings (Fig 1A, and S1 Table) revealed a significant reduction in ERG amplitudes in retinas with a 2.3-fold overexpression of ATF4 (Fig 1B and S1 Table). The AAV2/5-GFP-injected eyes showed a decline in ERG amplitudes similar to that found in control naïve animals with progressive ADRP. In addition to T17M, a slightly greater reduction was observed in the amplitudes of the scotopic ERG a- and b-waves in C57BL6 mice injected with AAV2/5-ATF4 (Fig 1B) suggesting that overexpression of ATF4 is harmful to both degenerating and normally functioning photoreceptors.

Accelerated loss of function is usually accompanied by a greater rate of cell death. Our examination of H&E-stained T17M retinal cryostat sections and counts of the number of photoreceptor rows (Fig 1C and 1D) revealed a significant decline in the number of rows of photoreceptor nuclei in T17M ATF4-injected retinas (6.9 ± 0.20 rows of nuclei in GFP-injected vs. 4.4 ± 0.09 in ATF4-injected retinas), which further supported the functional test results indicating that ATF4 overexpression in T17M retinas accelerates retinal degeneration. The C57BL6 retinas injected with AAV2/5 ATF4 also demonstrated photoreceptor cell death. We detected 10.33 ± 0.39 rows of photoreceptors vs. 12.85 ± 0.22 rows of photoreceptors in the control AAV2/5 GFP injected retinas.

It is known that the induction of ATF4 may result in increases in downstream targets [23]. Therefore, we further tested CHOP level in ATF4-overexpressed T17M retinas. Our data showed that ATF4 vector transduction resulted in the higher CHOP levels than those observed in AAV2/5-GFP-injected retinas (Fig 1B and S1 Table). This would indicate that the ATF4 viral transduction resulted in activation of the ATF4-CHOP pathway. Next, we were interested in whether photoreceptor cell death in ATF4-injected T17M retinas is associated with an increased level of caspase-3/7 activity and found the caspases’ activities to be highly upregulated in injected retinas (Fig 1E). Interestingly, the AAV2/5-ATF4 injection also resulted in elevated caspase-3/7 activity in wild type retinas. Thus, our data revealed that sustained ATF4 overexpression in photoreceptors provokes severe retinal degeneration in both wild-type and degenerating retinas through activation of apoptosis.

### T17M mice deficient in ATF4 manifest delay in the onset of retinal degeneration, as measured by functional vision test

We tested the hypothesis that a reduction in ATF4 would preserve visual function and prevent photoreceptor cell death in T17M retinas. For this we created an ADRP animal model with reduced ATF4 by crossing T17M^+/-^ *Rho*^+/+^ ATF4^+/-^ mice with *Rho*^-/-^ mice to obtain T17M^+/-^*Rho*^+/-^ATF4^+/-^ mice (henceforth referred to as T17M ATF4^+/-^or ADRP ATF4 deficient mice) and T17M^+/-^ *Rho*^+/-^ (henceforth referred to as T17M or ADRP) animals. These mice were compared to C57BL6 (*Rho*^+/+^) and ATF4^+/-^ (*Rho*^+/+^) mice. The ATF4^-/-^ mice develop severe microphthalmia with no recognizable lens, so they were unsuitable for this experiment. However, ATF4^+/-^ *Rho*^+/+^ mice (henceforth referred to as ATF4^+/-^ mice) appear to develop normally and have normal vision.

**Fig 2:**
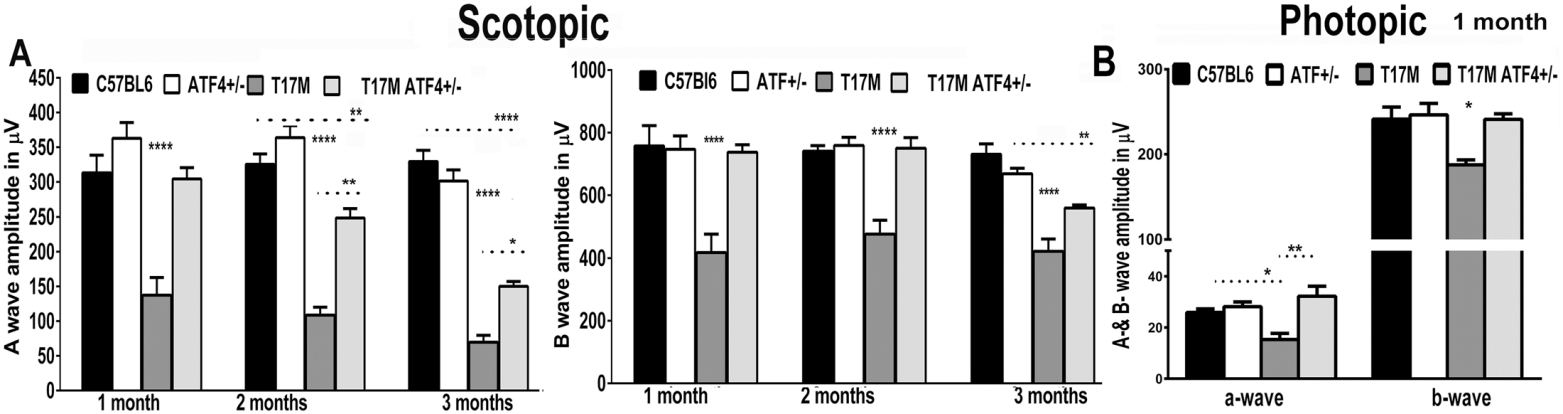
Knockdown of ATF4 prevents functional loss in T17M retina as measure by scotopic and photopic ERG. (**A and B**) ERG amplitudes were registered as described in the methods. The data are presented as mean ± SEM. See S1 Table and S2 Fig for details. **(A)** The two-way ANOVA analysis of the scotopic ERG results registered at 25 cd*s/m2 demonstrated no difference in the a-wave amplitudes of T17M ATF4^+/-^ mice (N = 10) at 1 month when compared to C57BL6 (N = 6) or ATF4 (N = 8) mice, whereas the T17M mice (N = 5) showed an ERG a-wave reduction (*P*<0.0001 as compared to all groups). However, a decline in the a-wave amplitudes began at 2 months in the T17M ATF4^+/-^ retina, with a subsequent decline at 3 months of age, when compared to C57BL6 retinas (*P*<0.01 and *P*<0.0001, respectively); but the T17M ATF4^+/-^ amplitudes were higher than those of 2- and 3-month-old T17M mice, (*P*<0.01 and *P*<0.05, respectively). The b-wave of the scotopic ERG amplitude was better preserved in the T17M ATF4^+/-^ retinas. No difference between T17M ATF4^+/-^ and C57BL6 or ATF4^+/-^ mice (n.s.) was detected during the first 2 months. By 3 months of age, the T17M ATF4^+/-^ started to exhibit a 24% decline in b-wave amplitudes as compared to C57BL6 retinas (*P*<0.01). (**B**) The photopic ERGs registered at 25 cd*s/m2, analyzed by one-way ANOVA, demonstrated dramatic declines in the a- and b-wave amplitudes in T17M retinas (N = 4) by 1 month compared to C57BL6 mice (N = 6) (*P*<0.05 for both waves). Knockdown of ATF4 in ADRP retinas (N = 4) significantly protected the T17M cone photoreceptors from functional loss and led to dramatic elevation of a-wave (*P*<0.01) and b-wave (*P*<0.05) amplitudes as compared to T17M retinas.

Analysis of the scotopic ERG responses (Fig 2A and S1 Table) demonstrated that whereas T17M mice experienced profound loss of a- and b-wave amplitudes at 1 month of age, the age-matched T17M ATF4^+/-^ animals demonstrated a functional protection of ADRP photoreceptors. No difference was observed between T17M ATF4^+/-^ and C57BL6 animals at this age. However, by 2 months of age, when T17M mice exhibited significant decreases in their ERG responses, T17M ATF4^+/-^ mice did show a slight drop in a-wave amplitudes, with b-wave amplitudes remaining unchanged relative to C57BL6 controls. At 3 months of age, the drop in ERG amplitudes for T17M ATF4^+/-^ mice became more noticeable. Significantly, despite the initial degeneration, the scotopic a- and b-wave ERG amplitudes were 2.15- and 1.3-fold greater, respectively, in T17M ATF4^+/-^ mice when compared to T17M animals (Fig 2A).

Light-adapted (photopic) ERG amplitude which was already diminished in 1-month-old T17M mice (Fig 2B and S1 Table) showed a remarkable recovery of both wave amplitudes in T17M ATF4 deficient animals. No difference was observed between 1-month-old T17M ATF4^+/-^ and C57BL6 or ATF4^+/-^ groups, suggesting normal cone function in P30 ATF4 deficient ADRP retinas. The functional test demonstrated that ATF4 deficiency in T17M significantly preserves vision in mice with retinal degeneration.

### T17M ATF4^+/-^ mice manifested delayed onset of retinal degeneration, as measured by imaging and histological analyses

**Fig 3:**
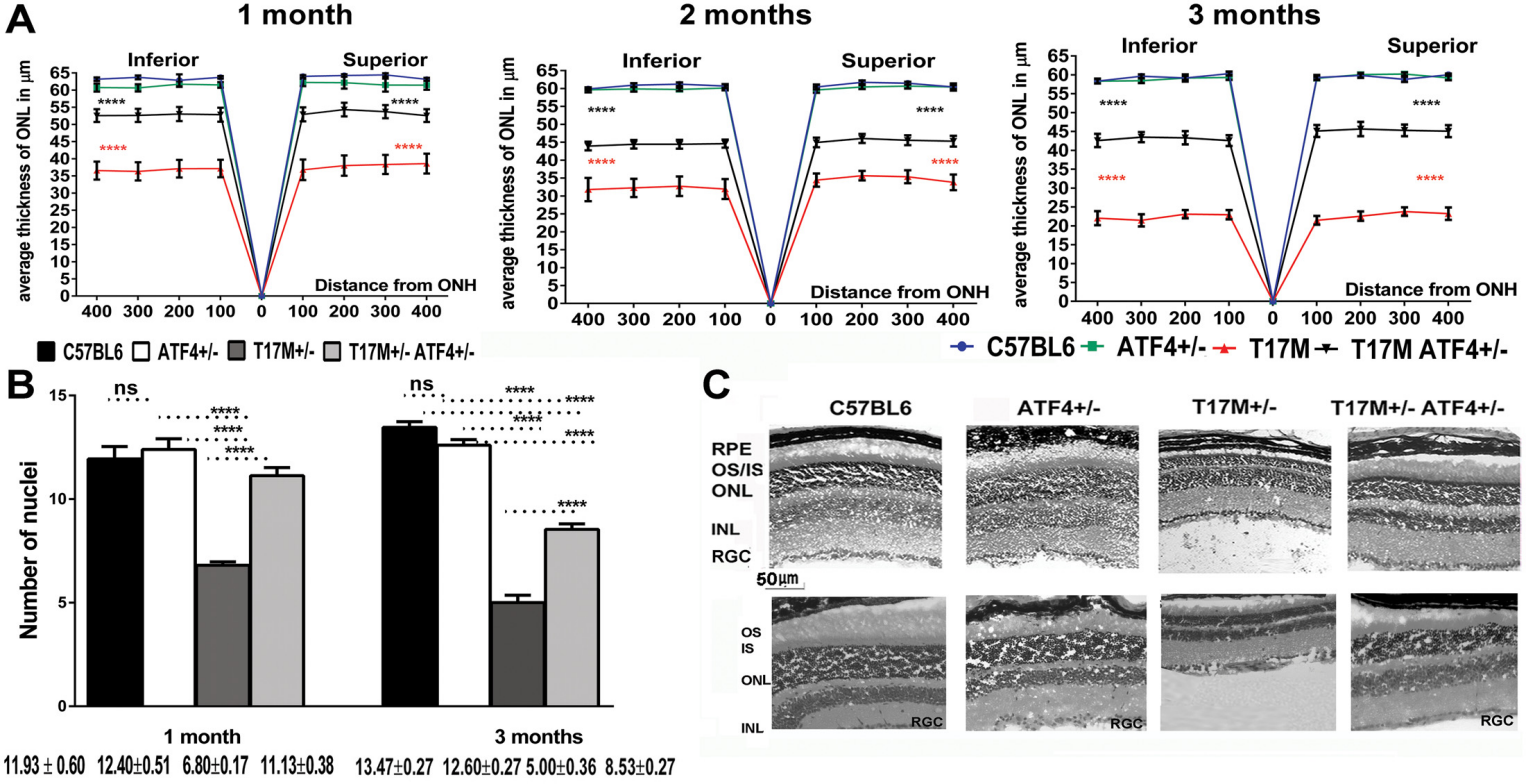
ATF4 knockdown protects T17M mice from loss of retinal integrity and photoreceptors. (**A)** We found dramatic increases in the average ONL thicknesses of both hemispheres of 1, 2, and 3-month-old T17M ATF4^+/-^ retinas compared to T17M mice (see also S1 Table). Representative spidergrams of the distribution of ONL thicknesses across the retina at 1, 2, and 3 months of age are shown. The data were analyzed by two-way ANOVA. The T17M ATF4^+/-^ retinas (N = 8) demonstrated an increase in the superior and inferior ONL thickness at 1, 2, and 3 months of age as compared to T17M mice (N = 11). All regions from both ADRP retinas were significantly different from C57BL6 (N = 7) and ATF4 (N = 7) mice (*P*<0.0001 for both strains and all regions). The data are presented as mean ± SEM. (**B**) The ATF4 deficiency in T17M mice protected their retinas from photoreceptor cell loss, resulting in an increase in the number of photoreceptors relative to T17M as measured by two way ANOVA (*P*<0.0001). The number of rows of photoreceptor nuclei between T17M ATF4^+/-^ (N = 4) and C57BL6 (N = 4) retinas was similar at P30, whereas the T17M mice lost 43% of their photoreceptor cells (*P*<0.0001 as compared to all groups). However, by 3 months, both the T17M ATF4^+/-^ and the T17M mice experienced a loss of photoreceptor cells as compared to C57BL6 mice (*P*<0.0001). The data are presented as mean ± SEM. **(C)** Representative images of H&E stained retinal sections from all four groups. Images of one-month-old (upper) and 3-month-old (bottom) control and experimental retinas. Scale bar indicates 50 μm.

We determined if ATF4 deficiency prevents loss of retinal integrity and photoreceptor cells in ADRP mice by analyzing the results from SD-OCT imaging and by histological evaluation of cryostat sectioned retinas stained with H&E. The SD-OCT measurements confirmed marked preservation of ONL thickness in 1-month-old T17M ATF4^+/-^ mice. Representative spidergrams of the distribution of ONL thicknesses across the retina at 1, 2, and 3 months of age are shown in Fig 3A and S1 Table. Representative SD-OCT images of one-month-old mice are shown in S2 Fig. The T17M retinas demonstrated a significant reduction in ONL thickness over a period of 3 months, whereas ATF4-deficient ADRP retinas showed a pronounced delay in this reduction of ONL thickness. Thus, at 1 month, the average T17M ATF4^+/-^ ONL thickness in the superior and inferior hemispheres were both appreciably preserved when compared to T17M mice and were 83% of the ONL thickness found in C57BL6 mice (S1 Table). The 2-month-old T17M ATF4^+/-^ mice also showed a significant increase in the average ONL thickness in both hemispheres when compared with T17M mice. This protective trend continued in 3-month-old T17M ATF4^+/-^ mice. Results from SD-OCT analysis of 3-month-old-retinas indicated that, despite a slight drop in the average inferior ONL thickness at 3 months (S1 Table), the average superior and inferior ONL thickness were both higher in T17M ATF4^+/-^ than in T17M animals (Fig 3A).

The finding of a marked preservation of retinal structure in T17M retinas deficient in ATF4 suggested that these animals were able to circumvent photoreceptor cell death. Histological analysis and counts of the number of photoreceptor nuclei rows in the retinas (Fig 3B and 3C) revealed a similar number of photoreceptor cells in T17M ATF4^+/-^ and C57BL6 retinas at P30, whereas T17M mice experienced a dramatic loss of photoreceptor cells. However, by P90, the T17M ATF4^+/-^ retinas showed a 37% loss of photoreceptor cells when compared to C57BL6, suggesting delayed retinal degeneration. This photoreceptor cell number was still notably higher than that of the degenerating P90 T17M retinas. Therefore, imaging and histological analyses demonstrated significant preservation of retinal structure and decreased photoreceptor cell death in T17M mice deficient in ATF4.

### The T17M ATF4^+/-^ mouse retinas demonstrate diminished ER stress response

**Fig 4:**
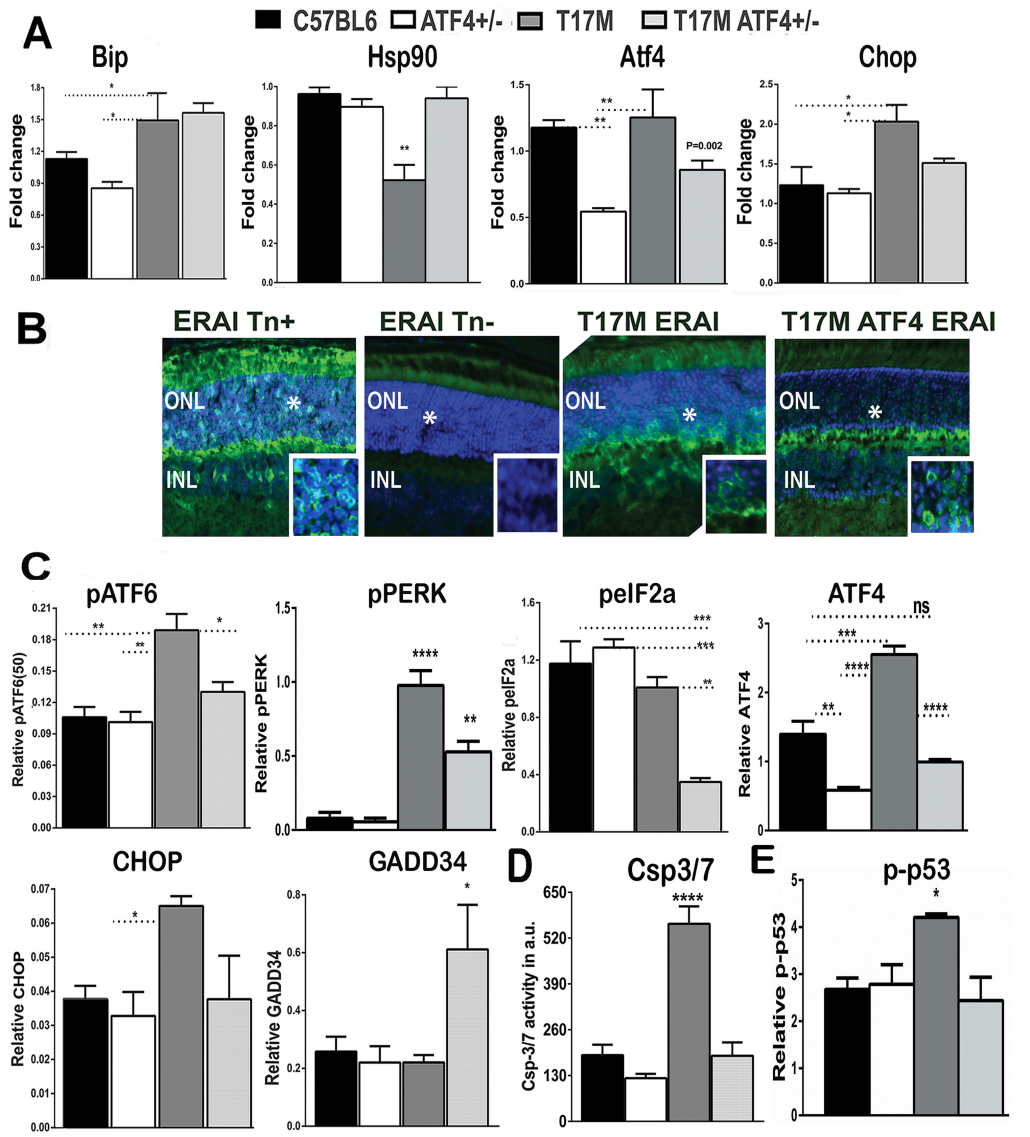
ATF4 knockdown in the ADRP retinas reduces overall ER stress response (N = 4). (**A**) The qRT-PCR data were analyzed by one-way ANOVA. *Bip*, *ATF4 and CHOP* mRNA expressions were elevated in P30 T17M retinas as compared to C57BL6 and ATF4 mice (*P*<0.05 for both). The ATF4-deficient ADRP retinas had a tendency to express less *Chop* mRNA as compared to T17M retinas. (*Hsp90* mRNA expression was significantly downregulated in T17M retinas (P<0.05 as compared to all groups). (**B**) The splicing of *XBP*1 mRNA resulted in immunohistochemical detection of GFP expression in the ONL of both T17M ERAI^+/-^ and T17M ATF4^+/-^ ERAI^+/-^ retinas at P15 and suggested activation of IRE1 UPR signaling in both ADRP groups. Tunicamycin-treated (ERAI Tn+) and–untreated (ERAI Tn-) ERAI retinas served as positive and negative controls, respectively. Locations of the inserts in the original images are shown with asterisks. (**C)** Western blot analysis revealed while T17M retinas demonstrated upregulation of pATF6 (50), pPERK, ATF4, and CHOP as compared to C57BL6 (*P*<0.01; *P*<0.0001; *P*<0.0001 and P<0.01 as compared to C57BL6 or ATF4, respectively), the ATF4 deficiency in these retinas led to downregulation of these UPR markers as compared to T17M mice (*P*<0.05; *P*<0.01; and *P*<0.001 respectively) and a trend toward downregulation for the CHOP protein. The T17M ATF4^+/-^ retinas were the only group with a significant decrease in peIF2a (P<0.01 as compared to T17M retinas) and increased GADD34 protein levels (*P*<0.05 for all groups). **(D)** Knockdown of ATF4 in ADRP retinas led to downregulation of caspase-3/7 activity. The T17M retinas demonstrated an almost 3-fold activation of caspase-3/7 (*P*<0.0001 as compared all four groups), whereas the T17M ATF4^+/-^ retinas were characterized by a level of caspase-3/7 activity comparable with wild type at P30. **(E)**Phosphorylated p53 was overexpressed in T17M retinas, as determined from protein extracts (P< 0.05 as compared to all groups). The data are presented as mean ± SEM. Representative Images of western blots treated with antibodies against cleaved pATF6, pPERK, peIF2a, ATF4, CHOP, GADD34, p-p53 and β-actin proteins are shown in S3 Fig. See also S1 Table for details.

Overexpression of ATF4, the UPR mediator, elevates production of CHOP and activates caspase-3/7 in injected retinas; therefore, ATF4 knockdown would be predicted to modulate UPR-associated hallmark expression as well as the status of executioner caspase activity in T17M mice. Fig 4 and S1 Table show the results of qRT-PCR, protein, and immunohistochemical analyses conducted in P15 and P30 retinas. These experiments showed elevated expression of *Bip* (1.3-fold) and *Chop* (1.65-fold) mRNAs, whereas *Hsp90* mRNA was downregulated (1.92-fold) in T17M retinas compared to wild type retinas, suggesting an augmented ER stress response. In T17M retinas deficient in ATF4, however, the *Hsp90* and *Chop* mRNA levels were restored to the level of wild type retinas. Both ADRP strain retinas showed activation of the IRE1 arm marker, with *Xbp1* mRNA splicing at P15, suggesting that UPR activation occurred earlier than at P30 (Fig 4A).

To show *XBP1* mRNA splicing, we generated T17M ERAI^+/-^ and T17M ATF4^+/-^ ERAI^+/-^ mice (Fig 4B), by cross breeding T17M and T17M ATF4^+/-^ mice with ERAI (ER stress-Associated Inducer) transgenic mice carrying a human *XBP*1 and a Venus (a variant of green fluorescent protein) fusion gene under control of a CAG promoter [24]. The splicing of *XBP*1 mRNA resulted in GFP expression in the ONL of both T17M and T17M ATF4^+/-^ retinas at P15, suggesting activation of IRE1 UPR signaling in both ADRP groups.

We also confirmed UPR activation by measuring the expression of UPR-induced cleavage of pATF6, pPERK, peIF2, ATF4, CHOP, and GADD34 proteins (Fig 4C and S1 Table). Protein analysis of these UPR markers demonstrated that the pPERK level was significantly upregulated in both ADRP retinas as compared to C57BL6 retinas. However, the pPERK upregulation was 2-fold lower in T17M mice deficient in ATF4 than in T17M mice. Analysis of other PERK signaling markers, the ATF4 and CHOP proteins, showed that they were up-regulated in T17M retinas and were significantly diminished in T17M ATF4^+/-^ retinas: ATF4 was up-regulated 1.8-fold in T17M retinas as compared to C57BL6 retinas and almost 2-fold downregulated in T17M ATF4^+/-^ retinas as compared to T17M retinas. The ATF4 level in T17M ATF4^+/-^ retinas was correspondingly compatible and higher when compared to C57BL6 and ATF4^+/-^ retinas.

We also found that levels of CHOP protein declined in T17M ATF4^+/-^ retinas, while its production was elevated 1.7-fold in T17M retinas when compared to C57BL6 mice. Upregulation of CHOP is known to control expression of GADD34 [25]. However, despite the downregulation of CHOP protein in ADRP retinas deficient in ATF4, the T17M ATF4^+/-^ mice continued to show increased GADD34 protein (3-fold increase in GADD34 protein). This result demonstrates that the ATF4 deficient T17M photoreceptors are able to transduce partially the signal for the sustained activation of GADD34 via CHOP-independent mechanism [26]. Because the GADD34 is known to provide regulatory feedback that can reverse translational attenuation, we next examined the peIF2a level. We found that the peIF2a was significantly downregulated in T17M ATF4^+/-^ mice. This unexpected response was not due to reduced eIF2a expression. In western blot analysis, we found no differences in the eIF2a level among all four groups (S3 Fig).

Activation of the ATF6 arm results in the cleavage of pATF6, which, together with the PERK pathway, contributes to CHOP overexpression. Our analysis of the cleaved pATF6 protein revealed that T17M mice had an almost 2-fold elevation of pATF6. In contrast, activation of the ATF6 UPR arm in P30 T17M ATF4^+/-^ retinas was significantly reduced (Fig 4C and S1 Table), and the level of cleaved pATF6 50 kD protein was comparable to that found in C57BL6 retinas. These data together with the reduction in peIFa and ATF4 in T17M ATF4^+/-^ retinas were in agreement with the observed downregulation of CHOP in T17M ATF4^+/-^ retinas.

ATF4 knockdown in T17M retinas also resulted in a large decrease in executioner caspase activity as compared to T17M retinas (Fig 4D and S1 Table). Notably, no differences were detected between T17M ATF4^+/-^ and C57BL6 retinas. Additionally, we found that downregulation of apoptotic markers in T17M ATF4^+/-^retinas coincided with a reduction in phosphorylated p53 (pp53) (Fig 4E), p53 is known to induce apoptosis [27].

Therefore, our results demonstrated that limiting ATF4 expression in T17M mice dramatically reduces overall ER stress response by attenuating the activation of the PERK and ATF6 arms and abolishing p53-dependent responses.

### The T17M ATF4^+/-^ mice demonstrate increases in NRF2 and diminished oxidative stress

**Fig 5:**
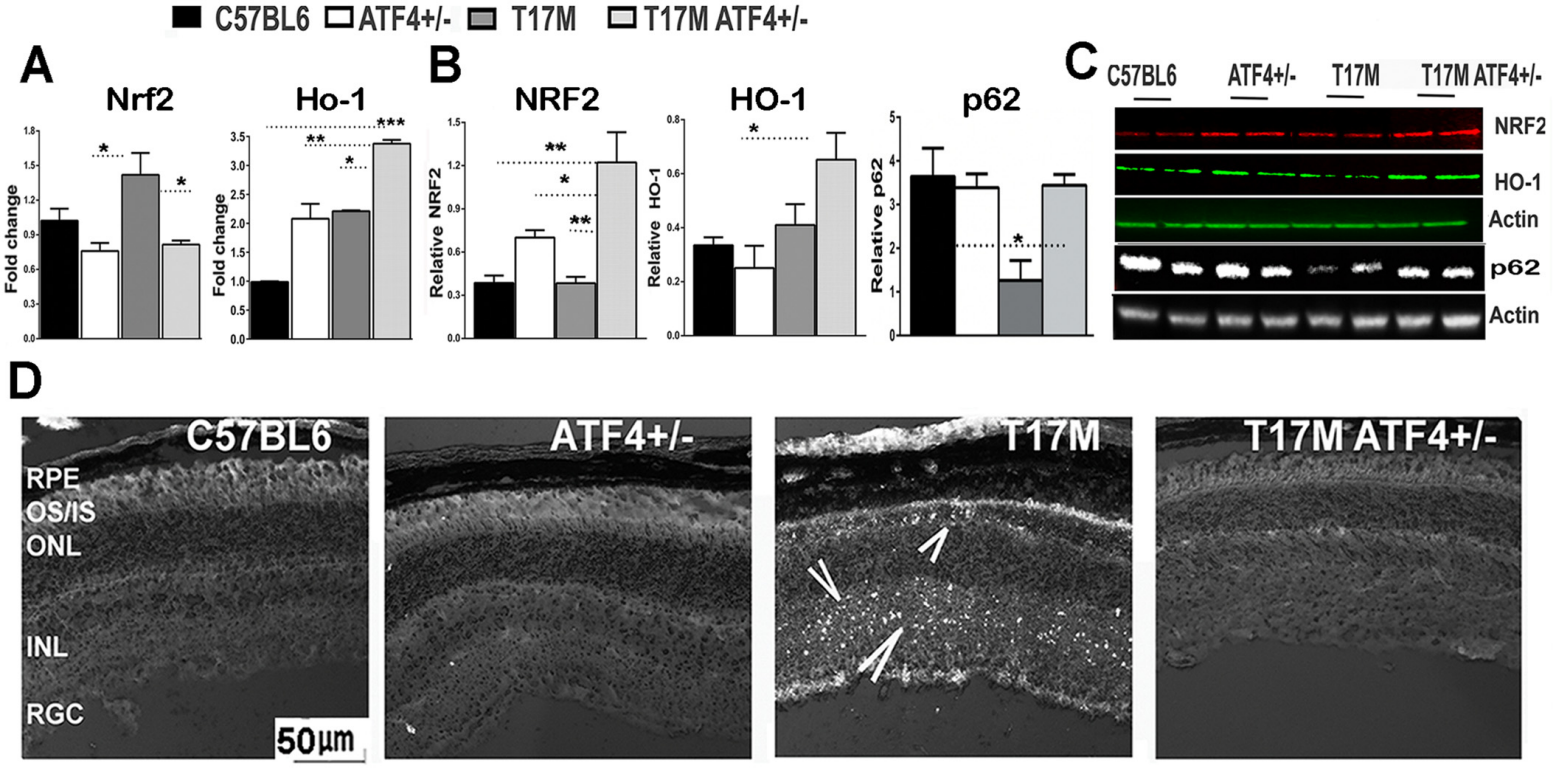
ATF4 knockdown launches the antioxidant cellular defense mechanism in P30 T17M and protects the ADRP retina against oxidative stress (N = 4). **(A)** *Nrf2* mRNA expressions were elevated in T17M retinas (*P*<0.05 as compared to all groups). But, *Ho-1* mRNA was only upregulated in T17M ATF4^+/-^ retinas (*P*<0.05; *P*<0.01 and *P*<0.001 when compared to T17M, ATF4^+/-^ and C57BL6 respectively). **(B)** Results of western blotting, analyzed by one way ANOVA, demonstrated that the antioxidant NRF2 and HO-1 expression was significantly up-regulated in T17M ATF4^+/-^ retinas as compared to C57BL6 retinas (*P*<0.01 and *P*<0.05, respectively) and T17M retinas (*P*<0.01 for NRF2). Expression of HO-1 protein in T17M ATF4^+/-^ had a tendency for up-regulation. In addition, p62 was significantly lower in T17M retinas as compared to all other groups (P<0.05). **(C)** Representative images of western blots treated with antibodies against p62, NFR2, HO-1, and β-actin proteins. **(D)** Oxidative stress was significantly diminished in the T17M ATF4^+/-^ retinas compared to T17M retinas with ongoing oxidative stress. Representative images of retinal cryostat sections stained with H2DCFDA. ROS positive cells are evident in the ONL and INL of the T17M retina and are indicated with arrows. The data are presented as mean ± SEM. See also for details S1 Table.

Knowing that NRF2 is a direct PERK substrate and that PERK-mediated activation of NRF2 can contribute to cell survival [28, 29], we next verified NRF2 expression level. Elevated pPERK in both P30 ADRP retinas suggested the activation of NRF2-induced antioxidant signaling in both ADRP retinas (Fig 5A, 5B and S1 Table). While *Nrf2* mRNA was significantly up-regulated in the P30 T17M retina, NRF2 protein level was similar to that found in the C57BL6 mice. In T17M ATF4^+/-^ retinas, western blot analysis revealed an increase in NRF2 protein at P30 (*P*<0.01) as compared to both the C57BL6 and T17M retinas. This was consistent with observed 1.5- and 3-fold up-regulation of the HO-1 mRNA and protein, respectively in T17M ATF4^+/-^ retinas (*P*<0.01 as compared to all groups). This data indicated a possible role of antioxidants in restoring a cellular homeostasis in the T17M ATF4^+/-^ retinas.

Analysis of T17M retinal protein extract at P15 (S1 Fig) revealed that the NRF2 level was indeed increased when compared to C57BL6 mice (*P*<0.01). This would suggest the induction of an inhibitory signal that prevented the elevated expression of NRF2 between P15 and P30. In addition to NRF2, we also observed a dramatic reduction in p62 levels in T17M retinas (Fig 5B and S1 Table) that is known to regulate KEAP1-ubiquitination at the posttranslational level [30] resulting in enhanced NRF2 degradation. Thus, the silencing of p62 has been previously shown to attenuate NRF2 activation [31] in cancer cells. Therefore, these posttranslational regulatory events seem to be involved in the pathogenesis of T17M retinas. Altogether, the activation of the UPR observed in our previous study[3] and increase in NRF2 at P15 (Fig 4B) as well as the PERK upregulation and diminishing in the NRF2 at P30 suggested a possible obstruction of the antioxidant program in T17M mice at later time points.

Consistently, we observed oxidative stress by performing immunohistochemical analysis and detection of reactive oxygen species (ROS) in retinal cryostat sections using a chloromethyl derivative of H2DCFDA fluorescence probe (Fig 5D). The T17M mice exhibited oxidative stress during ADRP, whereas the T17M ATF4^+/-^ retinas showed lack of ROS-positive cells.

### Retinal degeneration in T17M mice is associated with an impaired autophagy signaling

**Fig 6:**
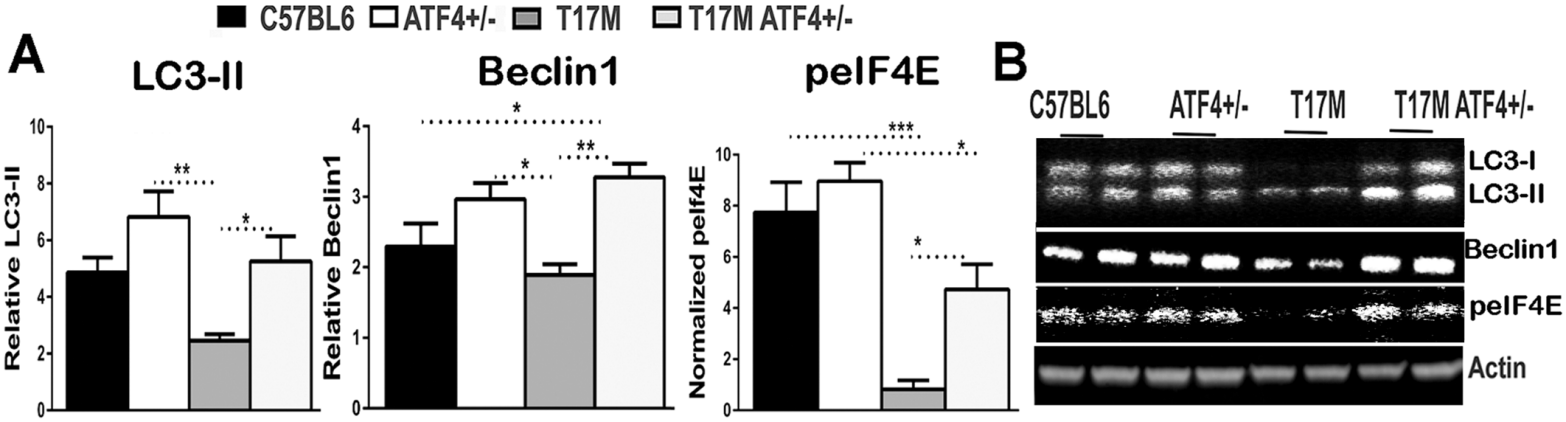
ATF4 deficiency in P30 T17M retinas results in upregulation of autophagosome associated genes. **(A)** Level of autophagy–related LC3-II (lipidated form) was diminished in T17M retinas as compared to C57BL6, ATF4^+/-^ and T17M ATF4^+/-^ retinas (*P* = 0.005, *P*<0.01 and *P*<0.05 respectively). Beclin-1 protein significantly up-regulated in T17M ATF4^+/-^ retinas as compared to C57BL6 and T17M retinas (*P*<0.05 and *P*<0.01 respectively). The T17M ATF4^+/-^retinas showed increased peIF4E protein levels as compared to T17M retinas (P<0.05). The data are presented as mean ± SEM. See also S1 Table for details.

It has been proposed that the Beclin-1 level, p62 degradation and the conversion of LC3 I to a lipidated form of LC3 II could serve as markers of autophagy [32, 33]. Moreover, p62 and Beclin-1 downregulation have been identified as markers of suppressed autophagy[34, 35]. Expression of p62 is required for the formation of autophagosomes through interaction with LC3 protein (MAP1LC3A in human) [36] that is in turn controlled by ATF4 [37, 38]. Therefore, after detection of diminished p62 level, we analyzed LC3 and Beclin-1 proteins. A significant reduction of LC3 II and Beclin-1 in the western blot analysis of T17M retinas as compared to all other groups, including the T17M ATF4^+/-^ mice was observed (*P*<0.05 and P<0.01) (Fig 6).

Recent study of selenite-induced UPR activation in Jurkat cells has proposed the role of p53-p38-MAPK-eIF4E-axis in ATF4 target selection response between apoptosis and autophagy [37]. The switch from apoptosis to autophagy in cells could occur by up-regulation of phosphorylated eIF4E. Therefore, we checked the peIF4E level in degenerating retinas and found an increase in the peIF4E protein in the T17M ATF4^+/-^ retinas as compared to T17M (*P*<0.05, Fig 6). The T17M retinas with up-regulated ATF4 level demonstrated significant inhibition of peIF4E which was likely regulated by activated p53 (Fig 4E).

### Delayed onset of retinal degeneration in T17M ATF4 mice is associated with improved RHO biosynthesis

**Fig 7:**
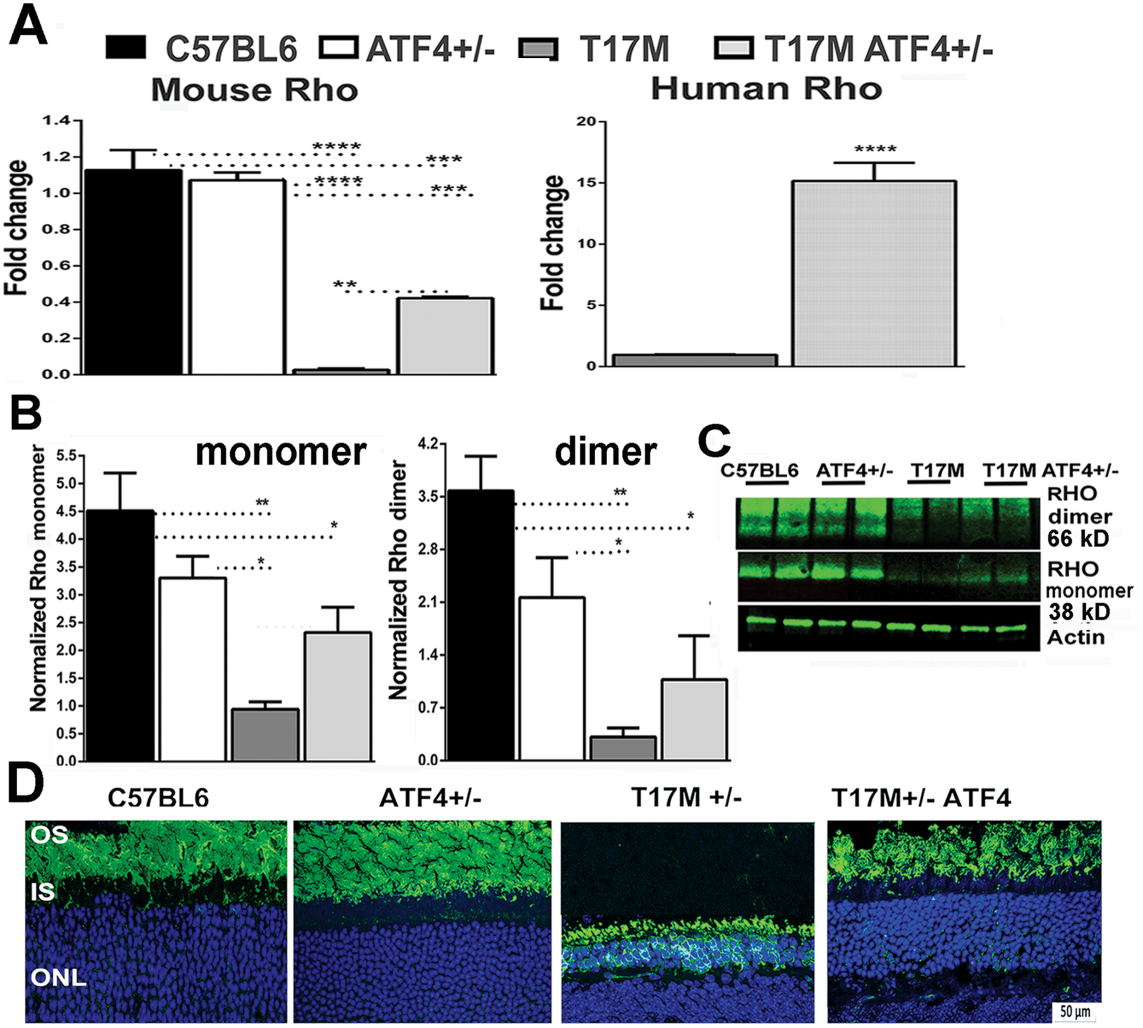
ATF4 knockdown in P30 T17M photoreceptors has a positive influence on the RHO expression machinery (N = 4 for all groups). (**A**) A significant decrease in the mouse RHO mRNA, analyzed by one way ANOVA, was found in the T17M retina as compared to C57BL6 and ATF4^+/-^ mice *(P*<0.001 for both). However, deficiency of ATF4 in these retinas led to a dramatic increase (16-fold) of mouse and human RHO mRNA expression as compared to T17M mice (*P*<0.01 and *P*<0.0001, respectively). (**B**) Increase in RHO mRNA led to elevated production of the RHO monomer and dimer in T17M ATF4^+/-^ retinas, whereas T17M mice experienced a dramatic (99% and 98%) loss of RHO production in photoreceptors, as measured by detection of dimer and monomer bands (*P*<0.01 for both bands as compared to C57BL6). (**C**) Representative images of western blots treated with antibodies against RHO. (**D**) Immunohistological analyses of P30 retinas with anti-RHO antibody (1D4, in green) revealed normal localization of rhodopsin in the T17M ATF4^+/-^ OS, compared with T17M mice with partially mislocalized RHO, suggesting improved clearance of RHO in the ADRP ATF4 deficient retina. ONL, outer nuclear layer; IS, Inner segments; OS, outer segments. Scale bar indicates 50 mm. The data are presented as mean ± SEM. See also S1 Table for details.

We further determined if ATF4 deficiency in ADRP retinas improves RHO expression and perhaps promotes degradation of misfolded or aggregated proteins in the cytosol. We performed a measurement of RHO mRNA and protein expression in all four groups of animals (Fig 7 and S1 Table). The T17M ATF4^+/-^ mice demonstrated enormous (16-fold) increase in expression of human and mouse Rho mRNAs (Fig 7A) and in total protein levels (Fig 7B) when compared to T17M retinas.

Enhancement of RHO production also correlated with the correct RHO localization within the rod photoreceptor (Fig 7D). Immunohistochemical analyses revealed that while T17M retinas experienced a mislocalization of a fraction of the RHO stained with the 1D4 antibody, no accumulation of RHO was detected within the ONL in T17M ATF4^+/-^ retinas. Clearance of trapped RHO was consistent with the activation of autophagy (Fig 6). Taken together, the present findings support a close link between retinal degeneration, ER stress, anti-oxidative defense, autophagy, and rhodopsin biosynthesis.

## Discussion

**Fig 8:**
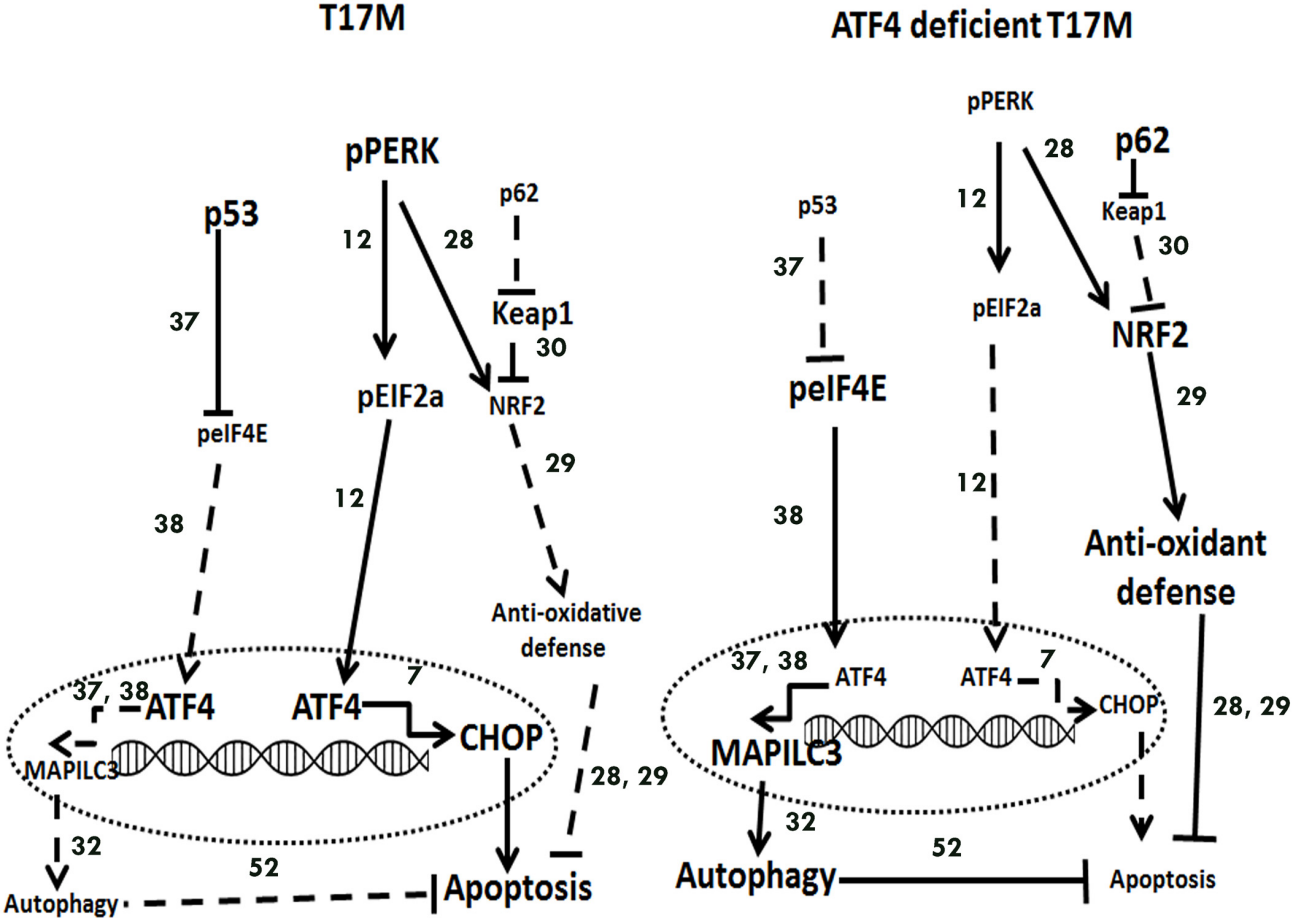
Proposed mechanisms of retinal degeneration in T17M and T17M ATF4^+/-^ mice. In T17M retinas, we observed up-regulation of pPERK, ATF4, CHOP, p53 proteins resulting in activation of apoptosis. In T17M retinas deficient in ATF4 we detected up-regulation of peIF4E, NRF2, GADD34 and autophagy genes resulting in delay of retinal degeneration. Increased and decreased levels of proteins in the retina are present as small and large fonts, correspondingly. Increased and decreased effects in solid and dashed lines, respectively are shown as well. Proposed in the literature links between signaling molecules are showed by numbers corresponding to their citations in the References section.

Elevation of ATF4, a mediator of PERK UPR signaling, accompanies progressive retinal degeneration [18]. However, the role of ATF4 in photoreceptor cellular pathology is unclear. Our study demonstrates that ATF4 plays a proapoptotic role during the ADRP progression associated with ER stress. Sustained overexpression of ATF4 is lethal as it leads to activation of executioner caspases resulting in photoreceptor cell death. Conversely, ATF4-deficiency in ADRP retinas results in a significant delay in the onset of retinal degeneration and increases photoreceptor survival. We speculate that the mechanism underlying the cytoprotection afforded by ATF4 deficiency in degenerating retinas, is linked to the p62, NRF2 and autophagy genes, which together orchestrate a synchronous reduction in ER stress, launch antioxidant defense, activate autophagy, and enhance rhodopsin biosynthesis as presented in Fig 8.

### Molecular mechanism of T17M photoreceptor degeneration

A persistently activated UPR in T17M mice is associated with severe retinal degeneration. Despite no detectable photoreceptor cell death at P15, our genetic approaches using T17M ERAI^+/-^ retinas indicate the activation of IRE1 arm at P15 and continued UPR activation.

The activation of PERK signaling in T17M retinas, detected in this study at P30 and in our previously conducted study at P15 [3], is associated with NRF2-dependent up-regulation of antioxidant defense at P15 (S1 Fig). Later, at P30, the observed down-regulation of p62 in these retinas may result in elevated KEAP1 activity that in turn leads to the degradation of excess NRF2 [30, 31]. The p62 has been previously shown to govern oxidant stress in cultured RPE and in the retinas of mice exposed to cigarette smoke [39]. This previous study also revealed that p62 silencing exacerbates the cigarette smoke-induced accumulation of damaged proteins, both by suppressing autophagy and by inhibiting NRF2 leading to increased protein oxidation. Therefore, the observed reduction of p62 in T17M retinas perhaps impedes the PERK-NRF2 anti-oxidant defense. Consequently, over the subsequent 2 weeks, the T17M retinas lose a great number of photoreceptors and experience rapid onset of retinal degeneration, resulting in a severe decline in both scotopic ERG amplitudes and photopic recordings. This implies that during the interval from P15 and P30, the T17M retinas experience rod cell death as well as the loss of cone cell function.

Activation of PERK signaling promotes ATF4 over-expression in T17M at P30 through uORF translation initiated by peIF2a. Phosphorylation (p) of eIF2α affecting translational initiation step is known to undergo dynamic changes due to persistently activated ATF4-CHOP-GADD34 negative feedback loop leading to dephosphorylation of peIF2a. Perhaps, because of this fact, we did not catch changes in peIF2a in T17M retinas at P30, while our previous study at P15 suggested changes in peIF2a [3]. Despite this fact, the PERK signaling markers PERK, ATF4 and CHOP are up-regulated in T17M retinas.

We showed, in a separate experiment, that ATF4 over-expression was cytotoxic for photoreceptors and induced severe retinal degeneration. Accelerated photoreceptor cell death and activation of cell death markers occur, respectively in T17M and the wild type mice following two weeks after AAV-mediated ATF4 over-expression. The decline in ERG amplitudes in naive T17M mice is due to a dramatic loss of photoreceptors and is associated with elevations of CHOP, p53 and activation of caspase3/7. We also found in these mice that a portion of total RHO was trapped in the ONL suggesting a role malfunctioned autophagosome-lysosome system in RHO clearance.

Therefore, persistent UPR, failure to augment antioxidant defenses, and autophagy occurring during stress appear to contribute to the pathogenesis of retinal degeneration in T17M mice. During ADRP progression, T17M mice experience a progressive loss of a- and b-wave scotopic ERG, which culminates in only 5 rows of photoreceptors remaining by 3 months of age.

### Molecular mechanism of T17M ATF4^+/-^ photoreceptor degeneration

The major contribution of ATF4 knockdown to the mechanism of retinal degeneration is likely through a reduction of overall ER stress response, induction of an antioxidant program, and restoring of autophagy, which together result in a delay of apoptotic photoreceptor cell death. The dramatic reduction in pPERK, pATF6, peIF2a, and CHOP proteins indicates a significantly lower degree of UPR activation in T17M ATF4^+/-^ retinas. The ATF4 level, as expected, is reduced in these animals and is undistinguishable from the level in C57BL6 mice. However, this level might be sufficient to overcome oxidative stress in degenerating retinas together with elevated NRF2 protein [13]. We speculate that in T17M ATF4^+/-^ retinas, despite the *Nrf2* mRNA level comparable to the C57BL6, the rise in NRF2 protein is most likely mediated by restoration of p62 levels. No significant differences in the mRNA copy number of *Nrf2* between the C57BL6 and T17M ATF4^+/-^ and remarkable elevation in the NRF2 protein levels perhaps indicate that posttranslational factors underpin the different levels of expression of NRF2 protein in the wild type and the ADRP retinas[40]. Thus, several factors have been found to increase the Nrf2 transcript level, including Nrf2 itself by autoactivation[41], and Jun[42]. The last one is known to be up-regulated in T17M mouse retinas[1]. The rise in NRF2 correlates with the PERK, which, despite its dramatic decrease in T17M ATF4^+/-^ as compared to T17M mice, may contribute to an NRF2-induced antioxidant defense.

p62 is one of the best known selective substrate of autophagy degradation. However, analysis of p62 and LC3-II conversion in both ADRP retinas suggests that the mechanism of p62 degradation is not strictly dependent on autophagy. Most likely the ubiquitin proteasomal system contributes to p62 degradation as well. Thus, the Parkin E3 ligase that ubiquitinates and degrades a diverse array of substrates, has been shown to degrade the p62 *in vi*vo [43]. Moreover, expression of Parkin is known to be ATF4 dependent [44]. Altogether this would imply an alternative mechanism for p62 degradation in T17M retinas.

Targeting of HSP90 during retinal degeneration has been proposed as a therapeutic strategy [45, 46]; however, the retinal degeneration in T17M retinas is associated with downregulation of *Hsp90* at P30 when compared to the wild type. This reduction could perhaps be responsible for the pp53 overexpression observed in T17M retinas since inhibition of HSP90 expression induces p53 over-expression [47]. Therefore, the observation that T17M ATF4^+/-^ mice show increases in *Hsp90* associated with the restoration of p53 to normal levels is a predictable outcome. This observation could perhaps relate to the proposed switch of ATF4-mediated apoptosis and autophagy during stress, since p53 appears to hold a core position in transducing the p38-promoted signal to either eIF2a or eIF4E [37]. Consequently, an increase in peIF4E and a decrease in peIF2a, as observed in ATF4 deficient T17M retinas, are perhaps associated with binding of ATF4 to the LC3 promoter and activation of autophagy as previously proposed [37]. Conversely, the T17M retinas experience impairment of autophagy activation associated with downregulation of *Hsp90* and p62, elevation of p53, reduction of peIF4E, and elevation of CHOP protein. Taken together, these findings support the notion that preferential activation of apoptosis vs. autophagy in T17M retinas, and alternatively, autophagy vs. apoptosis in T17M ATF4^+/-^ mice are important event in retinal degeneration. In support of this hypothesis, the UPR reduction due to knockdown of ATF4 in T17M mice correlates with normal levels of activated caspase-3/7 at P30. Perhaps, similar to targeting caspase-7 [1] and 12 [48], this approach is sufficient to delay vision loss in T17M mice.

Reduction in CHOP is associated with a paradoxical increase in GADD34, which, in turn, correlates with downregulation of peIF2a protein in T17M ATF4^+/-^ retinas. While GADD34 increase during CHOP downregulation in retinas requires further detailed study, the recent findings of a feedback loop provided by GADD34, canonical UPR signaling, and the enhancement of autophagy, known to be associated with GADD34 over-expression could in part explain our observation [49–51].

The analysis of T17M ATF4^+/-^ retinas revealed that both scotopic and photopic ERG amplitudes were preserved, and that cone photoreceptor function was well-maintained in 1-month-old animals. This may be due to the presence of normally functioning rod photoreceptors as reduction in ATF4 is known to reprogram the ER stress signaling network in ADRP retina. These results are of great importance as they support the notion that inherited retinal degeneration can be delayed. To the best of our knowledge, this is the first report to demonstrate rescue of rapidly degenerating ADRP retinas at P30 and a significant delay afterwards. The SD-OCT imaging shows a difference in average superior and inferior ONL thicknesses in the T17M ATF4^+/-^ mice, but this difference would appear to be due to altered photoreceptor morphology rather than a change in the number of photoreceptors. The H&E staining revealed no observable difference between C57BL6 and T17M ATF4^+/-^ mice, suggesting that no photoreceptor cell death occurred at P30.

### Rhodopsin expression machinery in ADRP retinas

The T17M ATF4^+/-^ retinas demonstrated elevated expression of both the mouse and human RHO mRNAs and increased RHO protein levels. Total RHO is also elevated in T17M ATF4^+/-^ retinas and reached the level about half that of RHO measured in C57BL6 mice (monomer). Moreover, this level corresponds to that found in RHO^+/-^ mice with normal vision that express 50% of the wild type RHO. Unlike the RHO^+/-^ mice, the T17M ATF4^+/-^ mice have both human and mouse RHO and the level of wild type rhodopsin may then be sufficient to maintain the function of photoreceptors during the first month of life. However, later on, the mutant rhodopsin transported to the outer segment could cause problems by reducing the ability to regenerate pigment after light exposure [52], thereby initiating the progression of retinal degeneration.

Activated autophagy in ATF4-deficent ADRP mice could be responsible for the clearance of partially accumulated RHO in the ONL of P30 retinas (Fig 7D). This finding correlates with the previously published data indicating that autophagy-dependent rhodopsin degradation prevents retinal degeneration [53] and that autophagy is essential to the long-term health of rod photoreceptors [54–56]. An increase in autophagy genes may also manifest temporal protection in T17M ATF4^+/-^ mice and these may contribute to photoreceptor health at early time points but may not be sufficient to maintain cellular homeostasis at later time-points.

Therefore, our results indicate an essential role for ATF4 in the induction of photoreceptor cell death. We can however propose that increased ATF4 expression during retina degeneration contributes to T17M photoreceptor cell death. Reduced ATF4 expression in degenerating retinas experiencing UPR activation, conversely, plays a pro-survival role, suggesting a therapeutic strategy for intervention in ADRP progression. ATF4 deficiency in the T17M retina under conditions of UPR activation, on the other hand, satisfies a photoreceptor cellular demand by adapting the UPR, overall reducing cellular stress in ADRP photoreceptors and activating autophagy that may clear a trapped rhodopsin in the ONL of ADRP retinas. Therefore, this signaling provides functional and morphological benefits to ADRP photoreceptors. This scenario is perhaps, sufficient to rescue the one-month-old ADRP retina and significantly delay further ADRP progression. Overall this indicates that PERK signaling in general and ATF4 in particular, is a target for delaying retinal degeneration in ADRP patients. Diminishing ATF4-dependent cellular stress leading to reduction of apoptosis and enhancement of autophagy may delay the onset of retinal degeneration. Based on our experimental data, reduction of ATF4 up to 50% is safe in T17M retinas. However, the therapeutic effect of ATF4 deficiency in other ADRP animal models requires further validation. This validation should also optimize the therapeutic dose at which ATF4 plays a protective role in degenerating retinas.

## Supporting Information

S1 Fig Expression of T17M RHO in ADRP rod photoreceptors results in photoreceptor cell death and leads to severe retinal degeneration. **(A)** Micron IV fluorescence image obtained from uninjected (left) and injected (right) with AAV2/5-GFP T17M retinas with fluorescence filter. Untransduced area in AAV2/5-GFP injected eye is shown with asterisk. **(B)** Analysis of H&E stained T17M retinal sections demonstrated dramatic photoreceptor cell loss that occurs between P15 and P30 (N = 4 for both, (*P*<0.0001). **(C)** Representative images of P15 C57BL6 and P15 and P30 T17M cryostat-sectioned retinas stained with H&E are shown on the side. **(D)** Expression of NRF2 in P15 T17M retinas. A western blot image is shown on a side. The data are presented as mean ± SEM.
(TIF)

S2 Fig Knockdown of ATF4 prevents loss of function and photoreceptor cell death in the one-, two-, or three-month-old T17M retinas. **(A)** Representative images of the scotopic and photopic ERG amplitudes registered at 25 cd*s/m2 for all four groups of animals. Please take in account that the scales (μV) for photopic ERG amplitudes are varied in all four groups of animals. For C57BL6 and ATF4 mice, the max amplitude is 300 and 200 μV respectively; for T17M is 150 μV, for T17M ATF4^+/-^ are 250 μV. **(B)** Representative SD-OCT images of retinas for all four groups of animals.
(TIF)

S3 Fig Representative images of western blots. Images were obtained by running retinal protein extracts from all four animal groups on polyacrylamide gels and probed with antibodies against the indicated proteins.
(TIF)

S1 Table Experimental data. Results of RNA, western blot, ERG, SD-OCT and histological analyses of the study.
(PDF)
