# Supplementary figures and images for: Limited ATF4 Expression in Degenerating Retinas with Ongoing ER Stress Promotes Photoreceptor Survival in a Mouse Model of Autosomal Dominant Retinitis Pigmentosa
Source: PLoS One. 2016 May 4;11(5):e0154779. doi: 10.1371/journal.pone.0154779 (PMC4856272; doi:10.1371/journal.pone.0154779)

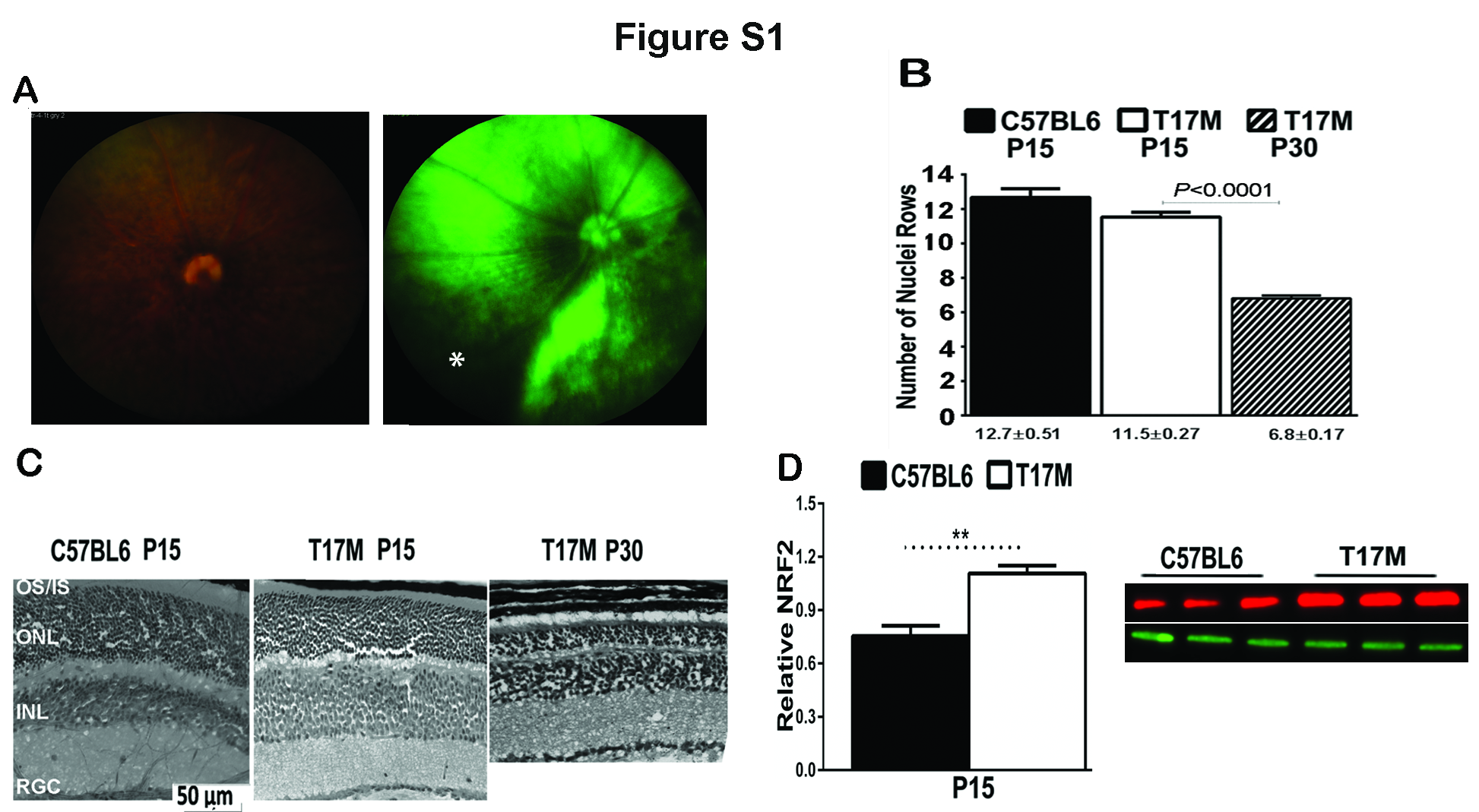

Supplement: S1 Fig — (A) Micron IV fluorescence image obtained from uninjected (left) and injected (right) with AAV2/5-GFP T17M retinas with fluorescence filter. Untransduced area in AAV2/5-GFP injected eye is shown with asterisk. (B) Analysis of H&E stained T17M retinal sections demonstrated dramatic photoreceptor cell loss that occurs between P15 and P30 (N = 4 for both, (P<0.0001). (C) Representative images of P15 C57BL6 and P15 and P30 T17M cryostat-sectioned retinas stained with H&E are shown on the side. (D) Expression of NRF2 in P15 T17M retinas. A western blot image is shown on a side. The data are presented as mean ± SEM. (TIF) [file pone.0154779.s001.tif]

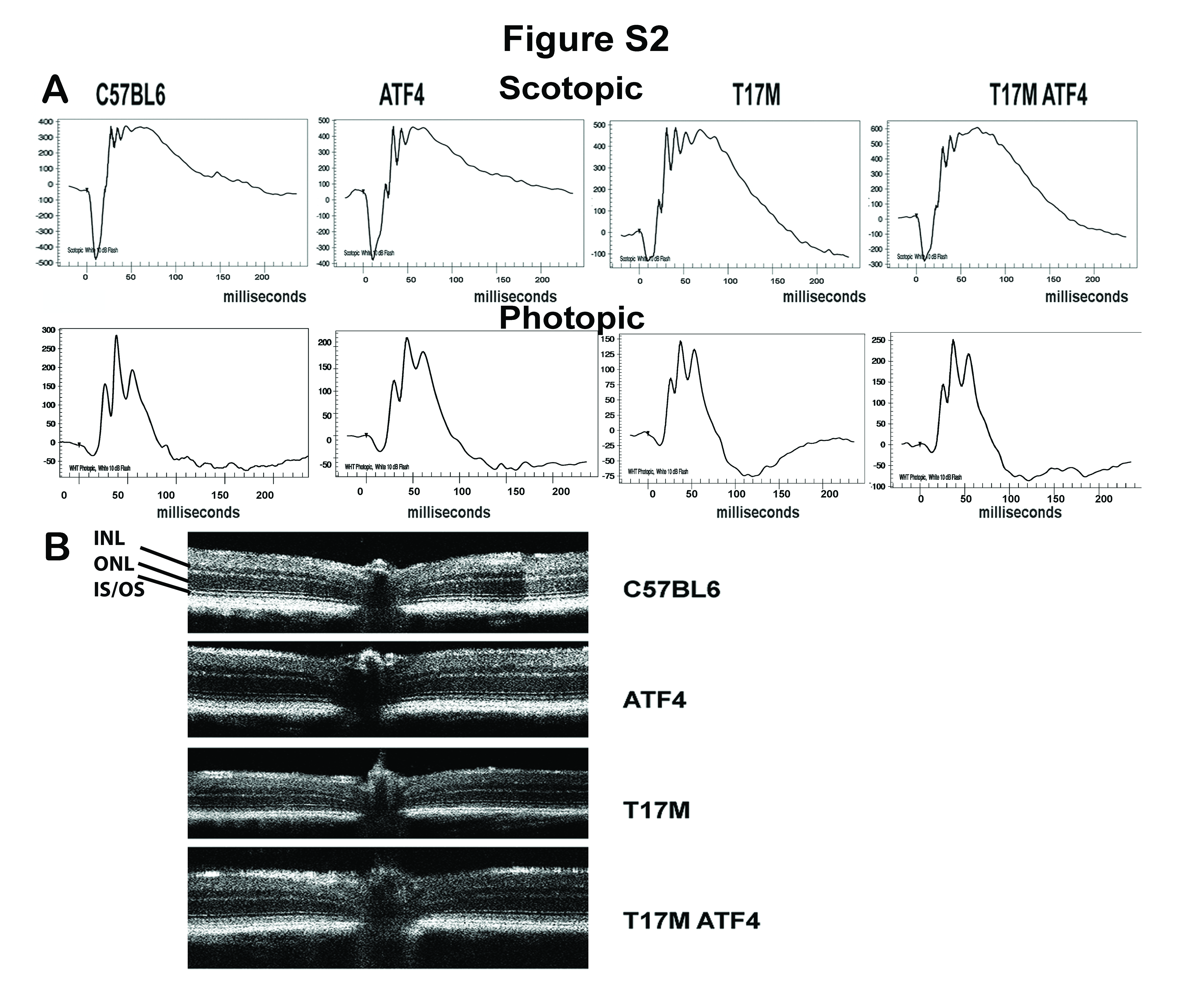

Supplement: S2 Fig — (A) Representative images of the scotopic and photopic ERG amplitudes registered at 25 cd*s/m2 for all four groups of animals. Please take in account that the scales (μV) for photopic ERG amplitudes are varied in all four groups of animals. For C57BL6 and ATF4 mice, the max amplitude is 300 and 200 μV respectively; for T17M is 150 μV, for T17M ATF4+/- are 250 μV. (B) Representative SD-OCT images of retinas for all four groups of animals. (TIF) [file pone.0154779.s002.tif]

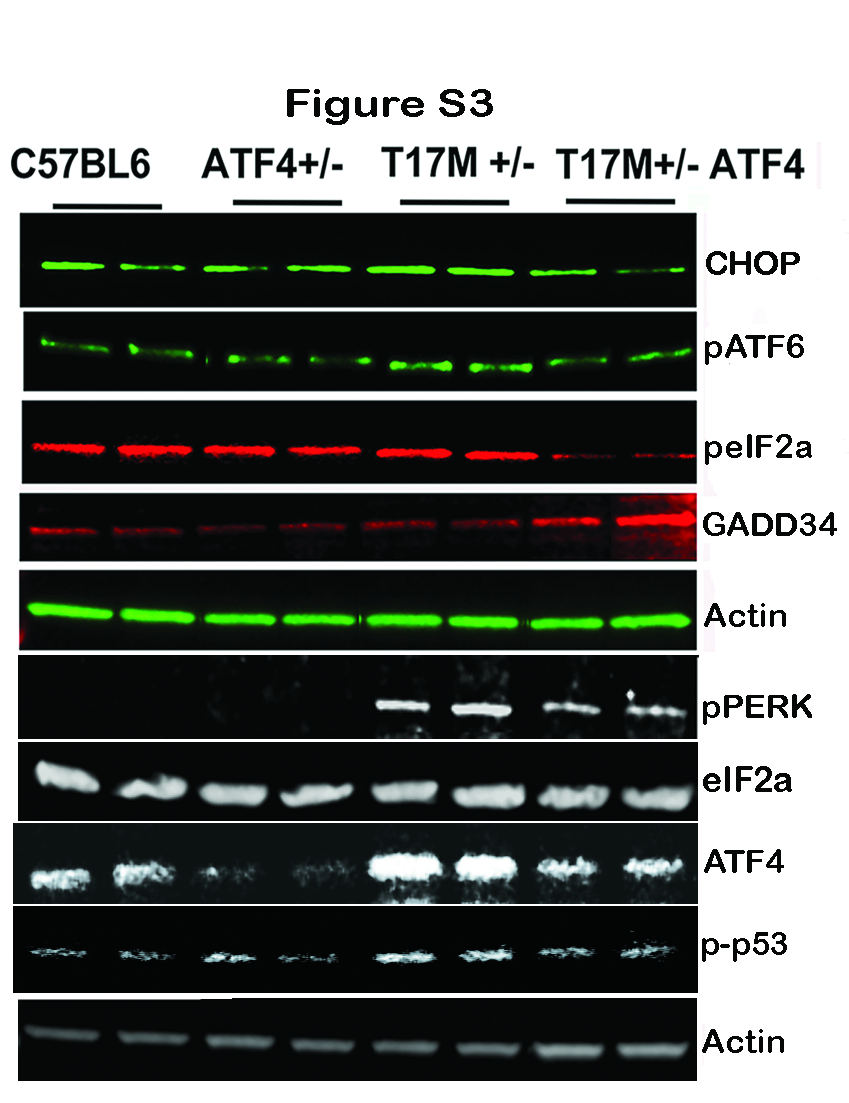

Supplement: S3 Fig — Images were obtained by running retinal protein extracts from all four animal groups on polyacrylamide gels and probed with antibodies against the indicated proteins. (TIF) [file pone.0154779.s003.tif]
