# Supplementary material for: Limited ATF4 Expression in Degenerating Retinas with Ongoing ER Stress Promotes Photoreceptor Survival in a Mouse Model of Autosomal Dominant Retinitis Pigmentosa
Source: PLoS One. 2016 May 4;11(5):e0154779. doi: 10.1371/journal.pone.0154779 (PMC4856272; doi:10.1371/journal.pone.0154779)
Supplement: S1 Table — Results of RNA, western blot, ERG, SD-OCT and histological analyses of the study. (PDF) [file pone.0154779.s004.pdf]

A. a- and b- wave amplitudes of the photopic and scotopic ERGs (in μV)

Over-expression of ATF4 by sub-retinal AAV2/5 delivery to P15 T17M RHO pups.

ERG amplitude	AAV2/5-ATF4	AAV2/5-GFP	Naïve
a-wave	76.06 ± 7.7	128.5 ± 13.83	137.4 ± 29.14
b-wave	339.5 ± 16.09	509 ± 19.17	492.2 ± 42.5

Over-expression of ATF4 by sub-retinal AAV2/5 delivery to P15 C57Bl6 pups.

ERG amplitude	AAV2/5-ATF4	AAV2/5 GFP	Naïve
a-wave	167.3 ± 39.28	394.5 ± 29.39	395.2 ± 19.02
b-wave	324.3 ± 93.92	686.5 ± 45.74	706.9 ± 48.57

B.

Western blot analysis of ATF4 over-expressed T17M retinas. : Normalized protein in a.u.

	T17M-AAV2/5-GFP	T17M-AAV2/5-ATF4
ATF4	0.204 ± 0.07	0.475 ± 0.05
CHOP	0.267 ± 0.06	0.497± 0.05

Caspase-3/7 activity

	T17M Injected with AAV2/5-ATF4	T17M Injected with AAV2/5-GFP	C57BL6 Injected with AAV2/5 -ATF4	C57BL6 Injected with AAV2/5 -GFP	Ratio: T17M injections ATF4/GFP	Ratio: C57BL6 Injections ATF4/GFP
Csp3/7	868.2 ± 40.6	576.6 ± 39.86	522.5±24.43	150.8±22.63	1.5	3.48

C.

a- and b- wave amplitudes of the photopic and scotopic ERGs (in μV)

Photopic ERG Amplitudes

Month	C57BL6	$ATF4^{+/-}$	T17M	T17M $ATF4^{+/-}$	Ratio: T17M/C57BL6	Ratio: T17MATF4$^{+/-}$ / T17M	Ratio: T17M ATF4$^{+/-}$ /C57Bl6
			a-wave				
1 month	27.04±1.72	28.13±1.87	15.33±2.39	28.94±2.73	0.567	1.89	1.07
			b-wave				
1 month	222.2±10.14	246.4±13.48	187.5±5.78	241.0±6.59	0.843	1.28	1.08

Scotopic ERG Amplitudes

Month	C57BL6	ATF4	T17M	T17M ATF4	Ratio T17M: C57BL6	Ratio T17M ATF4: T17M	Ratio T17MATF4: C57BL6
**a-wave**							
1	313±25.66	362.8±22.83	137.4±29.14	304.3±16.32	0.438	2.214	0.971
2	325.3±15.10	363.9±17.23	108.5±11.70	248.7±12.87	0.334	2.292	0.764
3	329.4±16.19	301.3±16.05	69.71±9.822	150.1±7.099	0.212	2.153	0.457
**b-wave**							
1	757.5±65.13	746.7±42.90	492.2±42.5	737.5±23.88	0.649	1.498	0.973
2	740.8±17.87	759.2±25.99	477.2±44.37	750.2±33.96	0.644	1.572	1.012
3	731.1±32.87	668.5±17.94	422±39.54	556.1±12.26	0.577	1.318	0.760

D. Superior and inferior ONL thickness

ONL thickness across the retina measured from optic nerve head (ONH)

Superior ONL Thickness	C57Bl6 (N=7)				ATF4 (N=7)				T17M (N=11)				T17M ATF4 (N=8)			
microns from ONH	100	200	300	400	100	200	300	400	100	200	300	400	100	200	300	400
P30	63.2± 0.49	63.7± 0.57	64.4± 0.47	63.1± 0.4	60.7± 1.16	60.6± 1.06	61.5± 1.89	61.4± 1.33	38.2± 1.47	37.8± 1.31	38.7± 1.22	38.6± 2.88	52.6± 1.85	52.6± 1.91	53.7± 1.92	52.8± 2.03
P60	60.4± 0.68	61.7± 0.48	61.5± 0.4	60.4± 0.55	59.6± 0.81	60.4± 0.55	60.7± 0.68	60.4± 0.98	34.3± 1.57	35.4± 1.15	35.1± 1.55	33.8± 2.2	43.9± 1.2	44.4± 1.19	45.6± 1.38	44.6± 1.11
P90	58.4± 0.63	59.6± 0.5	59.6± 0.5	60± 0.27	58.3± 0.59	58.5± 0.62	60.2± 0.41	59.2± 0.61	22± 1.86	21.5± 1.61	23.1± 1.09	23.2± 1.64	42.6± 1.75	43.5± 1.33	45.3± 1.54	42.6± 1.42

Inferior ONL Thickness	C57Bl6 (N=7)				ATF4 (N=7)				T17M (N=11)				T17M ATF4$^{+/-}$ (N=8)			
microns from ONH	100	200	300	400	100	200	300	400	100	200	300	400	100	200	300	400
P30	64± 0.38	64.3± 0.36	63.1± 0.4	63.7± 0.4	62.2± 1.08	62.2± 1.74	61.7± 0.8	61.5± 0.83	36.8± 3	38± 2.95	38.3± 2.78	38.7± 1.43	52.9± 2.04	54.3± 2	53± 2.05	52.6± 1.85
P60	60.4± 0.68	61.7± 0.48	61.5± 0.4	60.4± 0.55	59.6± 0.81	60.4± 0.55	60.7± 0.68	60.4± 0.98	34.4± 1.85	35.7± 1.31	35.4± 1.8	33.8± 1.84	44.9± 1.33	46± 1.26	44.4± 1.22	45.3± 1.85
P90	59.3± 0.66	59.8± 0.654	59.2± 0.93	60.3± 0.52	59.1± 0.38	60.1± 0.30	59.2± 0.48	59.3± 0.66	21.5± 1.16	22.6± 1.25	23.8± 1.11	22.9± 1.22	45.1± 1.59	45.6± 1.91	43.3± 1.74	45.1± 1.6

Average ONL thickness in superior and inferior retinas in microns measured by SD-OCT

Postnatal day	C57BL6	ATF4	T17M	T17M ATF4	Ratio T17M: C57BL6	Ratio T17M ATF4: T17M	Ratio T17MATF4: C57BL6
P30 Superior	63.4±0.209	61.2±0.274	38.3±0.218	52.8±0.102	0.604	1.378	0.832
P60 Superior	61±0.351	60.3±0.232	34.6±0.367	44.4±0.144	0.567	1.283	0.727
P90 Superior	59.4±0.416	58.8±0.252	22.4±0.384	43±0.237	0.377	1.919	0.723
P30 Inferior	64±0.288	61.8±0.218	37.9±0.4	53.4±0.396	0.592	1.408	0.834
P60 Inferior	61±0.351	60.3±0.232	34.8±0.428	45.5±0.232	0.570	1.307	0.745
P90 Inferior	59.5±0.279	59.6±0.29	22.8±0.495	45.3±0.128	0.381	1.986	0.761

E. UPR associated markers in P30 retinas. qRT-PCR: Gene expression, fold change

Genes	C57BL6	ATF4	T17M	T17M ATF4	Ratio T17M: C57BL6	Ratio T17M ATF4: T17M	Ratio T17MATF4: C57BL6
Bip	1.128±0.06	0.853±0.06	1.494±0.25	1.565±0.09	1.32	1.04	1.38
Hsp90	0.962 ± 0.03	0.896±0.03	0.522±0.07	0.94±0.05	0.54	1.8	0.97
Atf4	1.177±0.05	0.543±0.02	1.253±0.2	0.858±0.07	1.06	0.68	0.72
Chop	1.231±0.231	1.129±0.054	2.032±0.210	1.511±0.055	1.65	0.74	1.22

Western blot analysis: Normalized protein in arbitrary units (a.u)

Proteins	C57BL6	ATF4	T17M	T17M ATF4	Ratio T17M: C57BL6	Ratio T17M ATF4: T17M	Ratio T17M ATF4: C57BL6
pAtf6-50	0.106±0.01	0.101±0.010	0.189±0.01	0.130±0.00	1.787	0.687	1.229
pPERK	0.079±0.03	0.055±0.02	0.977±0.1	0.528±0.07	12.36	0.54	6.68
pEIF2	1.175±0.15	1.433±0.152	0.919±0.1	0.349±0.02	0.782	0.379	0.297
ATF4	1.4±0.19	0.584±0.04	2.55±0.12	0.993±0.03	1.82	0.38	0.7
CHOP	0.037±0.003	0.032±0.007	0.065±0.002	0.037±0.01	1.721	0.579	1.0
GADD34	0.258±0.05	0.220±0.05	0.220±0.02	0.611±0.15	0.880	2.911	2.571
p-p53	2.68±0.2	2.78±0.4	4.21±0.07	2.44±0.5	1.57	0.57	0.91

Caspase-3/7 activity: Luminescence in arbitrary units (a.u)

	C57BL6	ATF4	T17M	T17M ATF4	Ratio T17M: C57BL6	Ratio T17M ATF4: T17M	Ratio T17M ATF4: C57BL6
Csp3/7	188±29.59	122.5±12.26	560.8±49.42	186±37.96	2.982	0.332	0.989

F. Anti-oxidant proteins

qRT_PCR: Gene expression, fold change

Genes	C57BL6	ATF4	T17M	T17M ATF4	Ratio T17M: C57BL6	Ratio T17M ATF4: T17M	Ratio T17MATF4: C57BL6
NRF2	1.022±0.104	0.759±0.071	1.420±0.190	0.816±0.035	1.389	0.574	0.798
HO-1	1.000±0.000	1.579±0.252	2.210±0.014	3.377±0.064	2.210	1.528	3.378

Westrern blot analysis: Normalized protein in arbitrary units (a.u)

Proteins	C57BL6	ATF4	T17M	T17M ATF4	Ratio T17M: C57BL6	Ratio T17M ATF4: T17M	Ratio T17MATF4: C57BL6
p62	3.64±0.65	3.39±0.31	1.26±0.45	3.44±0.25	0.34	2.73	0.94
NRF2	0.386±0.05	0.701±0.05	0.384±0.04	1.22±0.21	0.99	3.18	3.16
HO-1	0.335±0.03	0.251±0.08	0.410±0.07	0.65±0.01	1.22	1.58	1.94

G. Autophagy Proteins

Western blot analysis: Normalized rhodopsin protein in arbitrary units (a.u)

Proteins	C57BL6	ATF4	T17M	T17M ATF4	Ratio T17M: C57BL6	Ratio T17M ATF4: T17M	Ratio T17MATF4: C57BL6
peIF4E	7.75±1.17	8.97±0.72	0.817±0.35	4.73±0.99	0.10	5.78	0.61
LC3-II	4.87±0.52	6.82±0.89	2.46±0.22	5.26±0.88	0.50	2.13	1.08
Beclin1	2.29±0.32	2.96±0.23	1.89±0.15	3.28±0.19	0.82	1.73	1.43

H. Rhodopsin and ER stress induced miRNA-708

qRT-PCR: Gene expression, fold change

Genes	C57BL6	ATF4	T17M	T17M ATF4	Ratio T17M: C57BL6	Ratio T17M ATF4: T17M	Ratio T17MATF4: C57BL6
Rho-M	1.127±0.111	1.072±0.043	0.027±0.008	0.422±0.009	0.024	15.856	0.375
Rho-H			0.949± 0.025	15.16± 0.746		16.12	
mRNA708	1.248±0.140	1.373±0.061	2.397±0.176	1.229±0.089	1.921	0.513	0.985

Western blot analysis: Normalized rhodopsin protein in arbitrary units (a.u)

RHO	C57BL6	ATF4	T17M	T17M ATF4	Ratio T17M: C57BL6	Ratio T17M ATF4: T17M	Ratio T17MATF4: C57BL6
Monomer	4.508±0.681	3.301±0.390	0.940±0.133	2.320±0.457	0.208	2.468	0.514
Dimer	3.577±0.457	2.162±0.528	0.314±0.117	1.075±0.580	0.087	3.423	0.300

I. List of antibodies and their dilutions

Protein	Company	Dilution
BIP	Santa Cruz	1:500
CHOP	Santa Cruz	1:500
peIF2α	Cell Signaling	1:1000
GADD34	Santa Cruz	1:500
pATF6 (cleaved)	Imaginex	1:500
Rhodopsin 1D4	University of British Columbia	1:1000
GFP	Abcam	1:400
ATF4	Cell Signaling	1:1000
pPERK	Cell Signaling	1:1000
p62	Cell Signaling	1:1000
Beclin1	Cell Signaling	1:1000
LC3-II	Cell Signaling	1:1000
NRF2	Santa Cruz	1:500
HO-1	Santa Cruz	1:500
p53	Cell Signaling	1:1000
peIF4E	Cell Signaling	1:1000
